# Pediatric emergency department visits for pedestrian and bicyclist injuries in the US

**DOI:** 10.1186/s40621-017-0128-5

**Published:** 2017-12-01

**Authors:** Katherine Wheeler-Martin, Stephen J. Mooney, David C. Lee, Andrew Rundle, Charles DiMaggio

**Affiliations:** 1 Department of Surgery, New York University School of Medicine, 550 First Avenue, NBV15N1, New York, NY 10016 USA; 2 Harborview Injury Research & Prevention Center, University of Washington, Seattle, WA USA; 3 Ronald O. Perelman Department of Emergency Medicine, New York University School of Medicine, New York, NY USA; 4 Department of Population Health, New York University School of Medicine, New York, NY USA

**Keywords:** Pedestrian, Bicycle, Emergency department, Traumatic brain injury, Youth, Pediatric

## Abstract

**Background:**

Despite reductions in youth pedestrian and bicyclist deaths over the past two decades, these injuries remain a substantial cause of morbidity and mortality for children and adolescents. There is a need for additional information on non-fatal pediatric pedestrian injuries and the role of traumatic brain injury (TBI), a leading cause of acquired disability.

**Methods:**

Using a multi-year national sample of emergency department (ED) records, we estimated annual motorized-vehicle related pediatric pedestrian and bicyclist (i.e. pedalcyclist) injury rates by age and region. We modeled in-hospital fatality risk controlling for age, gender, injury severity, TBI, and trauma center status.

**Results:**

ED visits for pediatric pedestrian injuries declined 19.3% (95% CI 16.8, 21.8) from 2006 to 2012, with the largest decreases in 5-to-9 year olds and 10-to-14 year olds. Case fatality rates also declined 14.0%. There was no significant change in bicyclist injury rates.

TBI was implicated in 6.7% (95% CI 6.3, 7.1) of all pedestrian and bicyclist injuries and 55.5% (95% CI 27.9, 83.1) of fatalities. Pedestrian ED visits were more likely to be fatal than bicyclist injuries (aOR = 2.4, 95% CI 2.3, 2.6), with significant additive interaction between pedestrian status and TBI.

**Conclusions:**

TBI in young pedestrian ED patients was associated with a higher risk of mortality compared to cyclists. There is a role for concurrent clinical focus on TBI recovery alongside ongoing efforts to mitigate and prevent motor vehicle crashes with pedestrians and bicyclists. Differences between youth pedestrian and cycling injury trends merit further exploration and localized analyses, with respect to behavior patterns and interventions. ED data captures a substantially larger number of pediatric pedestrian injuries compared to crash reports and can play a role in those analyses.

**Electronic supplementary material:**

The online version of this article (10.1186/s40621-017-0128-5) contains supplementary material, which is available to authorized users.

## Background

Youth pedestrian fatalities have continued to decline in recent years in the United States (US), (National Highway Traffic and Safety Administration 2017a) despite increases in adult pedestrian and bicyclist deaths between 2009 and 2015 (National Highway Traffic and Safety Administration 2017b). Still, motor vehicle crashes remain a primary killer of school-age children and teens, (Web-based injury statistics query and reporting system (WISQARS) 2016) and 21% of children killed in traffic crashes are pedestrians (National Highway Traffic and Safety Administration 2017a). Known risk factors for youth pedestrian injury include late afternoon and early evening activity/travel hours, high population density, traffic volume and vehicle speed, as well as on-street parking; furthermore, males, 5–9 year olds, and children living in low-income neighborhoods are disproportionately affected (Hotz et al. 2009). While most research has focused on fatalities, one study reported that 27% percent of children who survived pedestrian injuries went on to experience long-term physical sequelae, and 23% experienced psychological sequelae (Mayr et al. 2003). Parents of children injured by motor vehicles are likewise at risk for post-traumatic psychological symptomology (Spates et al. 2003).

Traumatic brain injury (TBI), a leading cause of injury death worldwide, (Carli and Orliaguet 2004) has been called “the most common cause of acquired disability in children,” (Patterson 1998) and is of particular concern in the youngest children (Bayreuther et al. 2009). Non-fatal outcomes include epilepsy (with the risk approaching 50% in children with direct injuries to brain tissue), (Lowenstein 2009) persisting neuropsychological deficits, (Fay et al. 1994) and impairments to executive function (Levin and Hanten 2005). While traumatic brain injury (TBI) is a clinically important feature of pedestrian and bicyclist injuries (Bruns and Hauser 2003) and motor vehicle crashes of all types are the leading cause of TBI-related death in the US, (Faul et al. 2010) there is less information available about the role of TBI in pediatric pedestrian and bicyclist injuries, including non-fatal injuries.

In previous analyses we have described national patterns of pediatric pedestrian and bicyclist crash-reported injuries (DiMaggio et al. 2016) as well as the ubiquity and clinical implications of pediatric TBI in New York state and New York City (DiMaggio 2013). In this study, we considered the descriptive epidemiology of pediatric emergency department trauma care for pedestrian and bicyclist injuries related to motor vehicles in the US, conducting a population-based analysis for the years 2006–2012 utilizing The Agency for Healthcare Research and Quality’s (AHRQ) Healthcare Cost and Utilization Project (HCUP) Nationwide Emergency Department Survey (NEDS) database. We sought to describe longitudinal trends in injury incidence nationally and regionally, exploring injury severity, prevalence of TBI, and case fatality for child and adolescent pedestrians compared with bicyclists.

## Methods

### Data sources

Data were obtained from NEDS for 2006–2012. Based on a 20% stratified single cluster sample of hospital-based emergency departments (EDs), NEDS is the largest most representative single publicly available ED database in the US. Core files consist of 100% of annual visits from sampled community hospitals, defined as non-federal, general, short-term, and specialty hospitals (such as pediatric hospitals and academic medical centers). NEDS utilizes a stratified sampling strategy based on geographic area, urban/rural area, ownership (i.e., government, private, not-for-profit), trauma center and teaching status, and bed size (Agency for Healthcare Research and Quality Healthcare Cost and Utilization Project (HCUP) 2016). For population-based rates, annual national and regional population estimates were obtained from HCUP, originating from the US Census Bureau. For hospital discharge-based rates, we used the universe of weighted NEDS ED visits. For travel-based rates, estimated national vehicle miles traveled were obtained from annual highway statistics maintained by the Federal Highway Administration (US Department of Transportation, Federal Highway Administration 2017).

### Inclusion criteria and study measures

We used R and MonetDB to read in the full initial dataset of 198,102,435 unweighted observations. Age groups were defined to be clinically relevant and consistent with available population estimates, as follows: 0–4, 5–9, 10–14, and 15–19 years. Four International Classification of Diseases, Ninth revision, Clinical Modification (ICD-9-CM) coded external cause of injury (E code) variables were used to identify injuries to pedestrians or pedalcyclists being struck by or involved in a collision with a motorized vehicle. We use the common term “bicyclist” throughout the remainder of this study to refer to all types of non-motorized pedalcyclists. We excluded E codes for bicyclists injuring other bicyclists or themselves (E8261–4, E8268–9), and injuries to drivers and motor vehicle occupants involved in a collision with a pedestrian (E8140–44). Pedestrians struck by bicyclists were included, given the potential resemblance in injury mechanism with other pedestrian-vehicle crashes. A detailed list of codes is available as an Additional file 1.

Injury severity, or probability of survival, was quantified using the ICD-derived Injury Severity Score (ICISS) as proposed by Osler et al. (Osler et al. 1996). First, survival risk ratios (SRRs) for each injury diagnosis were “...calculated as the ratio of the number of times a given ICD-9-CM code occurs in (surviving patients) to the total number of occurrences of that code.” Second, “the product of all the survival risk ratios (was computed) for each of an individual patient’s injuries” for up to ten different injuries (Segui-Gomez and Lopez-Valdes 2012). ICISS was then defined as the probability of patient survival and ranges from 0 to 1. As ICISS is, perhaps, most useful as a dichotomous indicator, (Stevenson et al. 2001) we defined severe injury as a survival probability <94% (i.e., a 6% or greater probability of death), as proposed by Gedeborg (Gedeborg 2009). In our previous analyses of trauma mortality, this cut point (0.94) produced a odds ratio of 6.75 (95% CI 6.48, 7.03) in multivariable logistic regression (DiMaggio et al. 2016).

Fatal outcomes were defined as patient records having a disposition of death in the ED or in the hospital, if admitted. Trauma center designations were based on the AHRQ “HOSP TRAUMA” indicator variable found in NEDS, which comes from the Trauma Information Exchange Program database (TIEP). Primary ICD-9-CM codes were categorized by the Barell Matrix, (Fingerhut et al. 2002; Barell et al. 2002) an injury diagnosis tool used internationally to standardize the classification of ICD-9-CM injury codes according to 12 nature-of-injury columns and 36 body-location rows. We also explored TBI severity using the 2002 Barell Matrix definition, as Type I (intercranial injury with moderate or prolonged loss of consciousness), Type II (no intercranial injury, loss of consciousness less than one hour), or Type III (no intercranial injury and no loss of consciousness).

### Statistical analysis

Descriptive epidemiology, including visit counts and population-based rates, age, gender, trauma center status, injury severity, and presence of TBI were estimated using survey-adjusted counts and means with the R package “sqlsurvey.” Ratio estimates and differences were calculated using the simulation method (Greenland 2004) based on survey-adjusted counts and standard errors, with each simulation consisting of 1000 random normal draws. Trends over time were evaluated with linear regression using year as the predictor variable.

Utilizing a retrospective cohort approach, we modeled the association of pedestrian vs. bicyclist injury with in-hospital fatality risk, controlling for age, gender, injury severity, trauma center status, and presence of TBI, with an interaction term for pedestrian status and TBI:

$$ \mathrm{death}={\upbeta}_{\mathrm{intercept}}+{\upbeta}_{\mathrm{age}}+{\upbeta}_{\mathrm{gender}}+{\upbeta}_{\mathrm{severity}}+{\upbeta}_{\mathrm{traumaCenter}}+{\upbeta}_{\mathrm{pedestrian}}+{\upbeta}_{\mathrm{TBI}}+{\upbeta}_{\mathrm{pedestrian}\cdotp \mathrm{TBI}} $$

Where, “age” was measured continuously in years, “gender” was an indicator variable for male (0) vs. female, “severity” was dichotomously coded 1 for ICISS <0.94 vs. ≥94 (0), “traumaCenter” was dichotomously coded 1 for treatment at a level 1 or 2 trauma center vs. non-trauma center (0), “pedestrian” was an indicator variable for pedestrian versus bicyclist (0), and TBI was dichotomously coded 1 for patients having traumatic brain injury as the primary diagnostic code per the Barell matrix, otherwise (0). The interaction term, “pedestrian^.^TBI” indicates the 4-level combination of the dichotomous variables for pedestrian status and TBI. Model and variable selection were based on descriptive results and univariable regression for the outcome of fatality. Modeling was conducted using the R “rms” package with robust covariances and options set to account for survey weighting and clustering. We tested for the assumption of linearity of the year variable and controlled for year-to-year variability in the survey results using an approach recommended by the Centers for Disease Control and Prevention (Centers for Disease Control and Prevention 2017; Bieler et al. 2010).

We assessed additive interaction of pedestrian status and TBI on fatality risk using a component-cause approach described by Darroch (Darroch 1997) and Rothman and Greenland (Rothman and Greenland 1998). To determine if the observed risk of pedestrian injury and TBI (R_ped + TBI+_) exceeds what we might expect if the two risks did not interact, we set up an interaction contrast (IC) as follows:

$$ \mathrm{IC}=\left({\mathrm{Risk}}_{\mathrm{ped}+\mathrm{TBI}+}-{\mathrm{Risk}}_{\mathrm{ped}-\mathrm{TBI}-}\right)-\left({\mathrm{Risk}}_{\mathrm{ped}+\mathrm{TBI}-}-{\mathrm{Risk}}_{\mathrm{ped}-\mathrm{TBI}-}\right)-\left({\mathrm{Risk}}_{\mathrm{ped}-\mathrm{TBI}+}\kern0.5em -\kern0.5em {\mathrm{Risk}}_{\mathrm{ped}-\mathrm{TBI}-}\right)={\mathrm{Risk}}_{\mathrm{ped}+\mathrm{TBI}+}\kern0.5em -{\mathrm{Risk}}_{\mathrm{ped}+\mathrm{TBI}-}\kern0.5em -{\mathrm{Risk}}_{\mathrm{ped}-\mathrm{TBI}+}\kern0.5em +{\mathrm{Risk}}_{\mathrm{ped}-\mathrm{TBI}-} $$

Interaction is considered present if IC ≠ 0, i.e., the combined effect does not equal the sum of the individual pedestrian and TBI effects. The proportion of excess risk attributable to the interaction between pedestrian injury and TBI was calculated as:

$$ \mathrm{IC}/{\mathrm{Risk}}_{\mathrm{ped}+\mathrm{TBI}+}. $$

We took into account survey variability by running bootstrap estimates of this equation and report the result as a point estimate with 95% confidence interval.

The study was approved by the New York University School of Medicine Institutional Review Board, and conforms to the STROBE statement for observational studies, excluding elements not applicable to a retrospective study design using administrative data. Detailed R code to reproduce or adapt our methods is available in Additional file 2. 

## Results

**Fig. 1:**
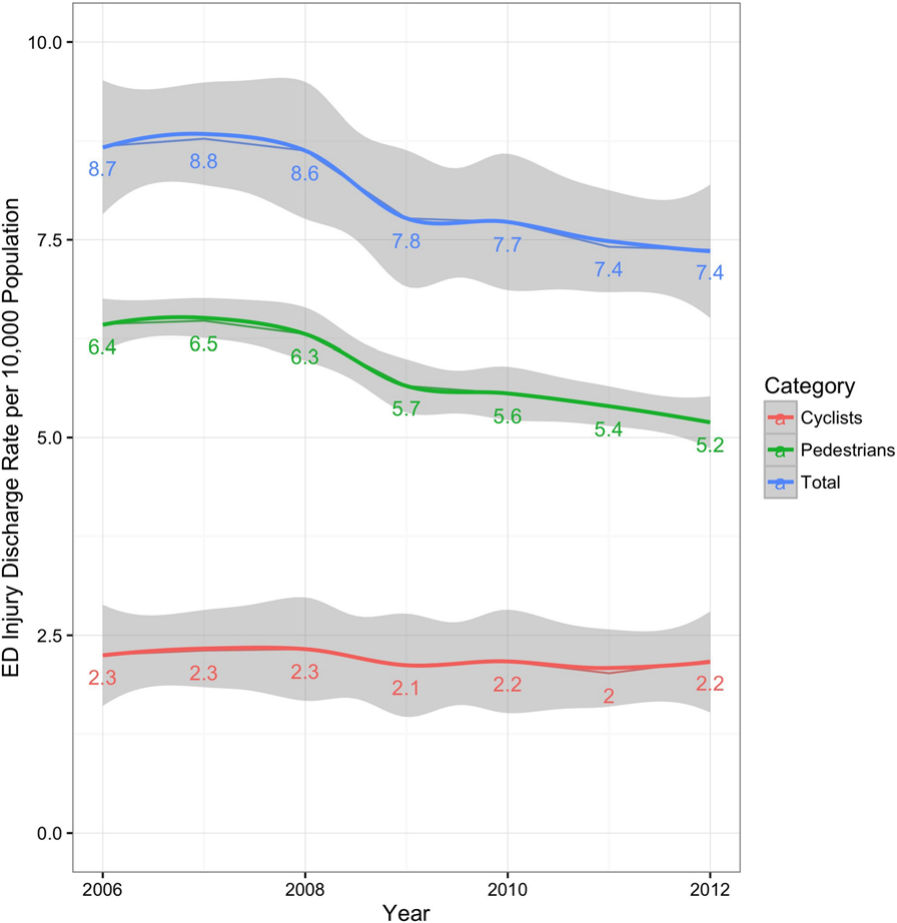
Pediatric pedestrian and bicyclist injury discharge rates per 10,000 population 0–19 year-olds, US EDs, 2006–2012

**Fig. 2:**
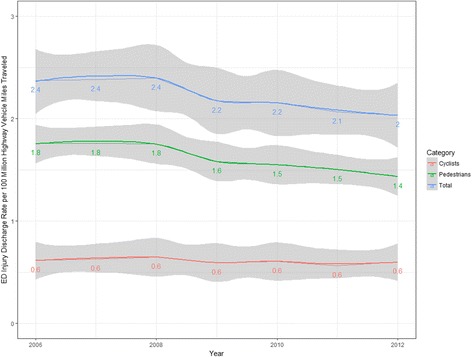
Pediatric pedestrian and bicyclist injury discharge rates per 100,000,000 highway vehicle miles, US EDs, 2006–2012

**Fig. 3:**
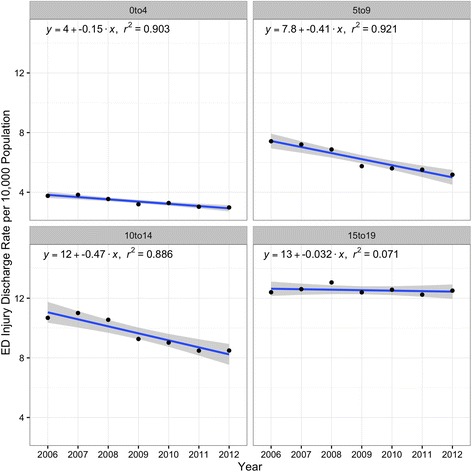
Age-group specific pediatric pedestrian and bicyclist injury discharge rates per 10,000 population, US EDs, 2006–2012

**Fig. 4:**
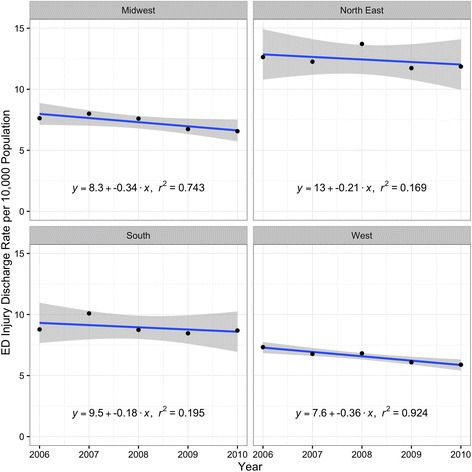
Region specific pediatric pedestrian and bicyclist injury discharge rates per 10,000 population, US EDs, 2006–2010

There were 217,551,676 (95% CI 217,496,020–217,607,332) survey-weighted total emergency department discharges for 0–19 year olds during the study period. A total of 467,093 (95% CI 464,110–470,076) of these discharges (0.21%, 95% CI 0.20, 0.21) were for pedestrian or bicyclist injuries related to motor vehicles, of which 72.7% (95% CI 72.6, 73.5) were pedestrians (Table 1). The pedestrian injury discharge rate declined 19.3% (95% CI 16.8, 21.9) whereas there was little or no change in the bicyclist injury rate (Fig. 1). A similar pattern was visible for travel-based injury rates per 100 million highway vehicle miles (Fig. 2). Declines in population-based pediatric pedestrian and bicyclist injury emergency department discharge rates were most notable for 5-to-9 year olds and 10-to-14 year olds (Fig. 3) and were steeper in the Midwest and West regions compared with the Northeast and South (Fig. 4). Females represented 40.0% (95% CI 39.6, 40.4) of pediatric pedestrians ED discharges, but only 19.6% (95% CI 19.0, 20.2) of injured bicyclists.

**Table 1:** Survey-weighted counts and standard errors (SE), US emergency department discharges for pediatric pedestrian and bicyclist injuries and deaths, ages 0–19 years, with annual national estimates of youth population and total vehicle miles traveled on US highways, 2006–2012. Discrepancies in totals (pedestrian plus bicycle) due to sampling variation and rounding

	Injuries			Deaths			US population estimate, 0–19 years	US highway vehicle miles traveled, millions
Year	Pedestrians (SE)	Bicyclists (SE)	Both (SE)	Pedestrians (SE)	Bicyclists (SE)	Both (SE)		
2006	52,961 (523)	18,547 (306)	71,501 (606)	515 (52)	85 (21)	600 (56)	82,482,488	3,012,888
2007	53,577 (516)	19,093 (306)	72,661 (600)	463 (53)	72 (18)	536 (56)	82,814,893	3,049,027
2008	52,417 (503)	19,326 (306)	71,729 (589)	511 (51)	92 (21)	603 (55)	83,236,036	2,992,705
2009	47,074 (487)	17,654 (301)	64,714 (572)	426 (45)	70 (20)	496 (50)	83,280,391	2,975,804
2010	46,268 (485)	18,055 (304)	64,307 (572)	410 (44)	58 (20)	468 (48)	83,118,264	2,985,854
2011	44,647 (465)	16,724 (283)	61,371 (544)	332 (41)	48 (15)	380 (44)	82,749,431	2,968,990
2012	42,839 (452)	17,982 (299)	60,811 (542)	386 (44)	63 (19)	449 (48)	82,324,415	2,988,021
Total	339,783 (1298)	127,379 (796)	467,093 (1522)	3042 (125)	488 (51)	3531 (135)	NA	NA

While the total ED-based visit rate of severely injured (ICISS <0.94) children and adolescents was 104.2 (95% CI 102.8, 105.6) per 10,000 pediatric ED discharges, the rate of severely injured pediatric pedestrians and bicyclists was 920.0 (95% CI 874.5, 965.5) per 10,000 pediatric ED discharges, or 8.8 (95% CI 7.9, 9.8) times greater. There was no meaningful difference in the ED-based discharge rates for severe injuries between pedestrians (946.0 per 10,000 discharges, 95% CI 892.9, 999.1) and bicyclists (919.5 per 10,000 discharges, 95% CI 801.3, 1037.7) and there was little or no change in these rates over the study period.

Between 2006 and 2012, there were 3531 (95% CI 3266–3796) pediatric pedestrian and bicyclist deaths, or 3.5% (95% CI 3.3, 3.7) of the total 101,147 (95% CI 99,742–102,552) deaths in children and adolescents based on ED data. Pedestrians accounted for 86.6% (95% CI 76.8, 96.4) of the total pedestrian and bicyclist decedents seen in an ED. There was a 24.8% (95% CI 17.8, 31.9) decline in the population-based rate of pediatric pedestrian and bicyclist ED discharge deaths from 7.3 (95% CI 5.9, 8.7) deaths per 1000,000 US 0–19 year olds in 2006 to 5.4 (95% CI 4.2, 6.6) in 2012. The case fatality rate for pedestrian and bicyclist ED discharges among 0–19 year olds decreased 12.0% (95% CI 4.5, 19.2) from 83.7 (95% CI 67.8, 99.6) deaths per 10,000 visits in 2006 to 73.7 (95% CI 58.6, 88.8) deaths per 10,000 visits in 2012.

Level 1 or 2 trauma centers cared for 69.2% (95% CI 62.9, 75.5) of all severely injured pediatric pedestrians and bicyclists in the US during the study period, with 15.0% (95% CI 14.0, 16.0) of all pediatric pedestrians and bicyclists discharged from level 1 or 2 trauma centers being severely injured, compared with 4.0% (95% CI 3.6, 4.4) of all pedestrians and bicyclists discharged from non-level 1 or 2 trauma centers. The proportion of all pediatric pedestrian and bicyclist deaths that occurred at level 1 or 2 trauma centers was 65.1% (95% CI 55.3, 74.9).

Lower extremity was the most common Barell matrix anatomic injury location, accounting for 45.0% (95% CI 43.4, 46.6) of all pediatric pedestrian and bicyclist ED discharges. There were 40 deaths among children and adolescents with a primary diagnosis of lower extremity injuries, which represented approximately 0.02% of such injuries. The next most common locations were “other” head, face and neck injuries (17.8%, 95% CI 17.0, 18.6) and injuries to the upper extremities (12.5%, 95% CI 11.9, 13.1).

Traumatic brain injuries (TBI) accounted for 7.6% of all bicyclist injuries (95% CI 6.2, 9.0) and 6.3% of all pedestrian injuries (95% CI 5.3, 7.4). Yet TBI was implicated in 55.5% (95% CI 27.9, 83.1) of pedestrian and bicyclist fatalities. The majority of TBI classified per the Barell matrix was “moderate” (67%) or “severe” (26%), involving intercranial injury and/or prolonged loss of consciousness, with severe TBI having the highest case fatality rate (Table 2). The unadjusted risk ratio for the association of TBI with fatality among injured pediatric pedestrian and bicyclists was 8.4 (95% CI 5.5, 11.2).

**Table 2:** Total pedestrian and bicyclist injuries, deaths, and case fatality rate with a primary diagnosis of traumatic brain injury (TBI), by grade (mild, moderate, or severe)

TBI Grade	Injuries (95% CI)	Deaths (95% CI)	Case Fatality Rate (%), (95% CI)
Type I (Severe)	6690 (6205–7175)	885 (553–1217)	13.2 (8.1–18.3)
Type II (Moderate)	17,290 (16037–18,543)	60 (38–83)	0.3 (0.0–2.1)
Type III (Mild)	1670 (1549–1800)	(NA)	(NA)

In a regression model comparing the risk of fatality for pedestrians versus bicyclists, controlling for age, gender, injury severity, and trauma center status, pediatric pedestrian injuries were associated with more than twice the risk of death as bicyclist injuries, with injury severity (by definition) and TBI being strong predictors of fatality (Table 3).

**Table 3:** Logistic regression model for pedestrian vs. bicyclist injury on the risk of fatality controlling for age, gender, injury severity, TBI, TBI*pedestrian status, and level 1 or 2 trauma center status; US emergency department discharges, 2006–2012

Variable	Adjusted Odds Ratio (95% CI)
Pedestrian	2.4 (2.3, 2.6)
Age	0.7 (0.7, 0.7)
Female	0.9 (0.9, 1.0)
Severe injury (ICISS < 0.94)	16.9 (15.9, 17.9)
TBI	6.3 (6.0, 6.6)
Trauma center	0.9 (0.9, 1.0)

The interaction contrast test was positive for additive interaction between pedestrian injury and TBI on fatality risk: IC = (4466.5–79.9) - (268.1–79.9) - (1968.3–79.9) = 2310 ≠ 0. Using bootstrapping methods to account for survey variability, we estimated that 49.1% (95% CI 38.4, 59.4) of the excess mortality in the group of pedestrians with TBI was attributable to the interaction between TBI and pedestrian injury.

## Discussion

The observed decline in US child and adolescent pedestrian ED injury discharges was consistent with published statistics, although there were more than twice as many motorized vehicle-related pedestrian and bicyclist ED injury discharges compared with police reported crash injuries (Web-based injury statistics query and reporting system (WISQARS) 2016). This was consistent with recent linkage analyses that have found fewer motor-vehicle traffic injuries in crash reports compared with hospital records, particularly in the case of less severe injuries, which may not be evident at the time of the crash nor reported to investigators (Watson et al. 2015; Conderino et al. 2017).

Although the early phase of the decline in pediatric pedestrian injuries reported in the 1990s was likely due to simultaneous declines in outdoor physical activity (Roberts 1993), in more recent years other factors may be at play, including interventions in the built environment such as Safe Routes to School, intended to safely increase rates of walking and biking to school in the US (DiMaggio et al. 2016). Indeed, school survey data indicate that rates of walking to school increased between 2007 and 2012 (US Department of Transportation, Federal Highway Administration 2013) while pediatric injury rates decreased. Although there was a decline in vehicle miles traveled from 2008 to 2010, the travel-adjusted rate of injury (Fig. 2) also declined. The growing prevalence of vehicle safety technology likely plays a role, with features such as backup cameras, automatic braking, and collision warning systems (Page et al. 2011; Mawson and Walley 2014). In 2014, NHTSA estimated that backup cameras alone could prevent over 95 deaths and 7000 injuries each year, when fully implemented (Barth 2014).

Our study found that pedestrian and bicyclist injuries are almost ten times more likely to involve severe injuries than any other ED visit among children, and that traumatic brain injury (TBI) was a strong, statistically significant predictor of in-hospital mortality in children hit by motor vehicles. In particular, pedestrian injuries in children and adolescents involving TBI were more likely to be fatal than bicycle-related TBI. These findings were consistent with several international studies documenting increased prevalence of severe TBI among pedestrians compared to cyclists or motor vehicle occupants (Javouhey et al. 2006; Majdan et al. 2013; Leijdesdorff et al. 2014; Cheng et al. 2017); uniquely, our study assessed mortality risk among young US pedestrians and bicyclists injured using a large, nationally representative source of ED records. The reasons for increased fatality risk among pedestrians with TBI is likely multi-faceted and future research may be able to shed light into these mechanisms. We speculate that increased severity of TBI in pedestrians may be an important explanatory factor, likely associated with the nature of crash impact and/or the protective effect of helmet use among bicyclists (Forsse et al. 2015; Macpherson and Spinks 2007).

Engineering, education, and enforcement interventions are well directed at preventing motor vehicle-related pedestrian crashes in the first place, yet concurrent research should also consider the secondary prevention of long-term sequelae from traumatic injuries when they do occur. Such research could involve measuring the impacts of aggressive, early interventions for TBI survivors or finding new treatment modalities to address long term physical, cognitive, and behavioral health effects. This is especially important in children, with growing evidence that moderate or even mild injury may result in “cognitive, personality and adaptive dysfunction (that) plague victims into maturity” (Parker 1994). Finally, the importance of optimal pre-hospital care and decision-making, such as preferential transport to trauma centers while minimizing transport times, has been well established, (Minardi and Crocco 2009) while choice of fluid resuscitation is an ongoing area of uncertainty and research (Chowdhury et al. 2014).

### Limitations

NEDS is a useful research tool, being the largest US all-payer ED database available to the public, containing a robust sample of nearly 30 million ED records each year. One feature of NEDS that may present limitations is that records are event-level, not patient-level. There are no patient identifiers to allow record linkage of multiple visits for the same condition. This limitation, however, is more pertinent to the study of chronic health conditions rather than acute injuries.

Specialized pediatric trauma centers are not identified in NEDS. For this reason we were unable to analyze individual levels of trauma center care or pediatric trauma care centers, although our analysis distinguished between trauma and non-trauma facilities. States, counties and zip codes are also suppressed in NEDS, thus geographic analysis in this study was limited to four regional designations: Midwest, Northeast, South, and West. Since our original purchase and analyses of NEDS, data has become available for 2013 and 2014. Additional analyses including these data would make for a stronger study, but must await our ability to fund further data purchases.

Despite its strengths as the largest representative sample of emergency department discharges in the United States, NEDS relies on the comprehensiveness of the data they receive from state agencies. While the AHRQ goes to great lengths to minimize missing data, some gaps are inevitable. We found the variables we analyzed to be generally complete. There were, for example, no missing data for age, primary diagnosis (and the variables that depended on it, like ICISS and Charlson score), and fatality status. Similarly, external cause of injury data were complete for the years reported (2009 to 2012). We found hospital trauma center status to be missing in 0.14% of observations. Information necessary to calculate Barell matrix designations was missing for 20.2% of entries. This last issue was not unexpected since the Barell matrix relies on a full 5-digit ICD-9-CM code and some of the codes were limited to 4 digits. We deferred using multiple imputation methods given the complex survey design and the number of records (millions) requiring computation power exceeding the capability of our research team. We instead restricted our statistics to full-case records with sensitivity analyses, recognizing the limitations of this approach.

AHRQ also notes that the inclusion of “outpatient surgery records that originate in the ED (e.g., fracture and dislocation procedures, appendectomies, etc.) can vary by state” (Agency for Healthcare Research and Quality Healthcare Cost and Utilization Project (HCUP) 2016). Finally, the NEDS sample does not include the growing number of free-standing urgent care centers or stand-alone emergency departments. Such centers, however, typically do not receive patients by ambulance with severe trauma or other life-threatening conditions. The displacement of pediatric patients with more minor injuries from hospital-based EDs to free-standing EDs could, however, impact the ED visit-based denominator of all injuries.

## Conclusion

Given their ubiquity and clinical implications, pediatric pedestrian and bicyclist injuries related to motor vehicles, particularly those resulting in TBI, remain critically important clinical and public health concerns. Differences between youth pedestrian and bicycling injury trends merit further exploration and localized analyses, with respect to behavior patterns and interventions. Emergency department data can be helpful in describing patterns in clinical outcomes of pediatric and adolescent injuries, and identify a broader spectrum of pedestrian and bicyclist injury victims than crash reports alone. In particular, this study highlights the impact of TBI on ED and hospital deaths of motor vehicle crash victims, especially young pedestrians. It suggests a role for concurrent clinical focus on TBI treatment and recovery alongside ongoing successful efforts across the US to reduce and prevent motor vehicle crashes with pedestrians and bicyclists.

## Additional files

Additional file 1: Detailed list of ICD-9-CM codes. (DOCX 13 kb)

Additional file 2: R code to reproduce or adapt study methods. (PDF 1207 kb)

## Abbreviations

AHRQ: Agency for healthcare research and quality
AP: Attributable proportion
CI: Confidence interval
ED: Emergency department
HCUP: Healthcare cost and utilization project
IC: Interaction contrast
ICD-9-CM: International classification of diseases, 9th revision, clinical modification
ICISS: International classification of diseases injury severity score
NEDS: Nationwide emergency department survey
OR: Odds ratio
SE: Standard error
SRR: Survival risk ratios
TBI: Traumatic brain injury
US: United States
