# Supplementary material for: Pediatric emergency department visits for pedestrian and bicyclist injuries in the US
Source: Inj Epidemiol. 2017 Dec 1;4:31. doi: 10.1186/s40621-017-0128-5 (PMC5709254; doi:10.1186/s40621-017-0128-5)
Supplement: Supplementary file 1 — Detailed list of ICD-9-CM codes. (DOCX 13 kb) [file 40621_2017_128_MOESM1_ESM.docx]

**Appendix**

Supplemental Table 1: Pedestrian-Injury Related E codes Included in the Definition of a Pedestrian Injured by Motorized and Other Vehicles

**E Code* Description­**

E8002 railway accident involving collision with rolling stock and injuring pedestrian

E8012 railway accident involving collision with other object and injuring pedestrian

E8022 railway accident involving derailment without antecedent collision injuring pedestrian

E8032 railway accident involving explosion, fire, or burning injuring pedestrian

E8042 fall in, on, or from railway train injuring pedestrian

E8052 pedestrian hit by rolling stock

E8062 other specified railway accident injuring pedestrian

E8072 railway accident of unspecified nature injuring pedestrian

E8107 motor vehicle traffic accident involving collision with train injuring pedestrian

E8117 motor vehicle traffic accident involving reentrant collision with another motor veh. injuring pedestrian

E8127 other motor vehicle traffic accident involving collision with motor vehicle injuring pedestrian

E8137 motor vehicle traffic accident involving collision with other vehicle injuring pedestrian

E8147 motor vehicle traffic accident involving collision with pedestrian injuring pedestrian

E8157 other motor vehicle traffic accident involving collision on the highway injuring pedestrian

E8167 motor vehicle traffic accident due to loss of control, without collision on the hwy, injuring ped.

E8177 noncollision motor vehicle traffic accident while boarding or alighting injuring pedestrian

E8187 other noncollision motor vehicle traffic accident injuring pedestrian

E8197 motor vehicle traffic accident of unspecified nature injuring pedestrian

E8207 nontraffic accident involving motor-driven snow vehicle injuring pedestrian

E8217 nontraffic accident involving other off-road motor vehicle injuring pedestrian

E8227 other motor vehicle nontraffic accident involving collision with moving object injuring pedestrian

E8237 other motor vehicle nontraffic accident involving collision with stationary object injuring ped.

E8247 other motor vehicle nontraffic accident while boarding and alighting injuring pedestrian

E8257 other motor vehicle nontraffic accident of other and unspecified nature injuring pedestrian

E8260 pedal cycle accident injuring pedestrian

E8270 animal-drawn vehicle accident injuring pedestrian

E8280 accident involving animal being ridden injuring pedestrian

E8290 other road vehicle accidents injuring pedestrian

* Pedestrian and cyclist E codes were based on AHRQ designations (https://www.hcup-us. ahrq.gov/db/tools/I9_Formats.TXT).

Supplemental Table 2: Bicycle-Injury Related E codes Included in the Definition of a Cyclist Injured by a Motorized Vehicle

**E Code* Description**

E8003 railway accident involving collision with rolling stock and injuring pedal cyclist

E8023 railway accident involving derailment without antecedent collision injuring pedal cyclist

E8033 railway accident involving explosion, fire, or burning injuring pedal cyclist

E8043 fall in, on, or from railway train injuring pedal cyclist

E8053 pedal cyclist hit by rolling stock

E8063 other specified railway accident injuring pedal cyclist

E8073 railway accident of unspecified nature injuring pedal cyclist

E8106 motor vehicle traffic accident involving collision with train injuring pedal cyclist

E8116 motor vehicle traffic accident involving reentrant collision with another motor veh. injuring cyclist

E8126 other motor vehicle traffic accident involving collision with motor vehicle injuring pedal cyclist

E8136 motor vehicle traffic accident involving collision with other vehicle injuring pedal cyclist

E8146 motor vehicle traffic accident involving collision with pedestrian injuring pedal cyclist

E8156 other motor vehicle traffic accident involving collision on the highway injuring pedal cyclist

E8166 motor vehicle traffic accident due to loss of control, without collision on the hwy, injuring cyclist

E8176 noncollision motor vehicle traffic accident while boarding or alighting injuring pedal cyclist

E8186 other noncollision motor vehicle traffic accident injuring pedal cyclist

E8196 motor vehicle traffic accident of unspecified nature injuring pedal cyclist

E8206 nontraffic accident involving motor driven snow vehicle injuring pedal cyclist

E8216 nontraffic accident involving other off road motor vehicle injuring pedal cyclist

E8226 other motor vehicle nontraffic accident involving collision with moving object injuring pedal cyclist

E8236 other motor vehicle nontraffic accident involving collision with stationary object injuring cyclist

E8246 other motor vehicle nontraffic accident while boarding and alighting injuring pedal cyclist

E8256 other motor vehicle nontraffic accident of other and unspecified nature injuring pedal cyclist

* Pedestrian and cyclist E codes were based on AHRQ designations (https://www.hcup-us. ahrq.gov/db/tools/I9_Formats.TXT).
