# Supplementary material for: Pediatric emergency department visits for pedestrian and bicyclist injuries in the US
Source: Inj Epidemiol. 2017 Dec 1;4:31. doi: 10.1186/s40621-017-0128-5 (PMC5709254; doi:10.1186/s40621-017-0128-5)
Supplement: Supplementary file 2 — R code to reproduce or adapt study methods. (PDF 1207 kb) [file 40621_2017_128_MOESM2_ESM.pdf]

R Code to Accompany Emergency Department Discharges for Motor-Vehicle Related Pediatric and Adolescent Pedestrian and Cyclist Injuries in the United States

Charles DiMaggio
Bellevue - NYU Trauma Service

April 3, 2017

Contents

1 Load Files **2**

2 Methods **3**
2.1 Data 3
2.2 Analyses 4

3 Descriptive Epidemiology **6**

4 Injury Severity **23**

5 Deaths **25**

6 Trauma Centers **30**

7 Categories and Causes **32**

8 Regression **38**
8.1 Interaction on Additive Scale 42

9 Appendix **45**
9.1 Pedestrian and Cyclist Ecodes 45
9.2 R Syntax to Create Pedestrian and Cyclist Variables 47

1 Load Files

Establish a connection to the out-of-memory Monet database containing the NEDS injury file. Check the file and fields. Create a sqlsurvey object from the MonetDB injury table.[1] Source and create a dplyr table from the MonetDB file.

```
# connect MonetDB
neds <- dbConnect(MonetDBLite(),"~/NEDSMonet")
dbListTables(neds)

dbRemoveTable(neds, "neds0612"/"neds_0612")

dbListFields(n,eds, "neds_0612")
db,GetQuery(neds, "SELE,CT COUNT(*) FROM ned,s_0612") # 198,102,435

# check data types
dbGetQuery(neds, "SELECT c.id, c.name, c.type, c.type_digits
    FROM sys.columns c LEFT JOIN sys.tables t ON c.table_id = t.id
    WHERE t.name = 'neds_0612';")

# print out a few observations of a variable
dbGetQuery(neds,
"SELECT traumacenter FROM neds_0612 LIMIT 10;"
)

# sqlsurvey object from  MonetDB table.
# identify factors
fax<-c("ecode1", "ecode2", "ecode3", "ecode4", "hcupfile", "agegrp", "isrcodedescr",
"isrsitedescr", "isrsite2descr", "isrsite3descr","agecat", "agecat_kids", "ped_cycl_mvc",
"ped_mvc", "cycl_mvc","region")

monInj<-sqlsurvey(id="key_ed",strata="neds_stratum",weights="discwt",key="id",
check.factors=fax, database="/Volumes/Promise Pegasus/DATA/HCUP/NEDSMonet",
driver=MonetDBLite(),table.name="neds_0612")

# connect database to R via dplyr
NEDScon <- src_monetdb(embedded="~/NEDSMonet")
NEDStab<- tbl(NEDScon,"neds_0612")
glimpse(NEDStab)
summarise(NEDStab, mean_age=mean(age)) # 38.35852
count(NEDStab,drug_or_alcohol) # 7,372,821
count(NEDStab,traumacenter)
count(NEDStab,region)
```

Write simulation functions for differences (declines) using survey results.

[1]See http://rpackages.ianhowson.com/rforge/sqlsurvey/ and http://rpackages.ianhowson.com/rforge/sqlsurvey/man/sqlsurvey.html

```
sim.mean.diff <- function(m1, sd1, m2, sd2) {
    est = mean((rnorm(n = 1000, mean = m1, sd = sd1) - rnorm(n = 1000,
        mean = m2, sd = sd2))/(rnorm(n = 1000, mean = m1, sd = sd1))) *
        100
    print(est)
}


sim.sd.diff <- function(m1, sd1, m2, sd2) {
    est = sd((rnorm(n = 1000, mean = m1, sd = sd1) - rnorm(n = 1000,
        mean = m2, sd = sd2))/(rnorm(n = 1000, mean = m1, sd = sd1))) *
        100
    print(est)
}
```

2 Methods

2.1 Data

Data were obtained from the US Agency for Healthcare Research and Quality (AHRQ) HCUP NEDS for the years 2006 to 2012. HCUP is a group of inpatient and outpatient files created by AHRQ. Based on a 20% stratified single-cluster sample of hospital-based EDs, NEDS is the largest most representative single publicly available ED database in the US. Core files consist of 100% of visits from sampled hospitals and contain approximately 66% of all ED visits for that year. Sampled hospitals are non-federal general and specialty hospitals including public hospitals and academic medical centers. Stratification variables include geographic area, urban/rural, ownership, trauma center and teaching status, and bed size.[2]

Comma-separated text core files for each year were read into an R data-frame, which were then converted to MonetDB column-oriented database files and appended to each other using the R packages "DBI"[3] and "MonetDBLite"[4], resulting in a full dataset of 198,102,435 unweighted observations. Listed ages were categorized into 4 pediatric age groups: 0-4, 5-9, 10-14, 15-19. Age groups were defined to be both clinically relevant, and consistent with available population estimates to allow the calculation of population-based rates. Four

[2]Introduction to the Healthcare Cost and Utilization Project's (HCUP) Nationwide Emergency Department Sample (NEDS), 2013. Agency for Healthcare Research and Quality. https://www.hcup-us.ahrq.gov/db/nation/neds/NEDS2013Introduction.pdf. Published Dec, 2015. Accessed Sept 20, 2016.

[3]R Special Interest Group on Databases (R-SIG-DB), Hadley Wickham and Kirill Müller (2016). DBI: R Database Interface. R package version 0.4-1. https://CRAN.R-project.org/package=DBI

[4]Hannes Muehleisen, Anthony Damico, Mark Raasveldt, Thomas Lumley and MonetDB Development Team (2016). MonetDBLite: In-Process Version of MonetDB for R. R package version 0.3.1. https://CRAN.R-project.org/package=MonetDBLite

external cause of injury variables[5] were searched to identify injuries to pedestrians and/or bicyclists injured by being struck or involved in a collision with a moving motorized vehicle. Ecodes for pedal cyclists injuring other individuals or themselves (E8261-4, E8268-9) and ecodes denoting injuries to drivers or other individuals involved in a collision with a pedestrian (E8140-44) were excluded. A detailed list of codes is available as an appendix.

Injury severity was quantified using the ICD-derived Injury Severity Score (ICISS) as proposed by Osler et al as a means of estimating injury severity using ICD codes in administratively collected hospital discharge data.[6] ICISS is calculated in two steps. First, survival risk ratios (SRRs) for each injury diagnosis in a data set are "...calculated as the ratio of the number of times a given ICD-9 code occurs in (surviving patients) to the total number of occurrences of that code". Second, the ICISS for an individual patient is calculated as "the product of all the survival risk ratios for each of an individual patient's injuries (for as many as ten different injuries)."[7] The ICISS is then defined as the probability of patient surviving their injuries and ranges from 0 to 1. An ICISS cut-off of less than 0.94 was used to categorize patients into those with the most severe injuries as proposed by Gedeborg.[8] This indicator variable identifies patients with a 6% or greater probability of dying, and has performed well in previous analyses, returning an odds ratio of 6.75 (95% CI 6.48, 7.03) in multivariate logistic regression analysis of trauma mortality.[9] Fatalities included those prior to and after hospital admission. Trauma center designations were based on the AHRQ "HOSP_TRAUMA" indicator variable found in the NEDS hospital file for that year. Primary ICD-9 codes were categorized by the Barell Matrix, an injury diagnosis tool used internationally to standardize the classification of ICD-9 injury codes 800 to 995 according to 12 nature-of-injury columns and 36 body-location rows. [10]

2.2 Analyses

The descriptive epidemiology, including visit counts and population-based rates, ages, genders, types of injuries and external causes of injury and costs for alcohol and substance-related emergency department traumatic injury dischages was assessed using survey-adjusted counts

[5]American Medical Association. International classification of diseases, 9th revision, clinical modification: physician ICD-9-CM, 2005. Chicago, IL: AMA Press; 2004.

[6]Osler T, Rutledge R, Deis J, Bedrick E. ICISS: an international classification of disease-9 based injury severity score. J Trauma. 1996; 41(3):380-8.

[7]Seguí-Gómez M, Lopez-Valdes FJ. Injury Severity Scaling. In: Li G, Baker SP, ed. Injury Research. New York, NY: Springer; 2012: 291.

[8]Gedeborg R. Injury incidence over time based on hospital admissions filtering by thresholds, risk categories or indicator diagnoses. Joint meeting of international collaborative effort on injury statistics and the global burden of disease-injury expert group. National Center for Health Statistics. Centers for Disease Control and Prevention. http://www.cdc.gov/nchs/injury/ice/boston2009/boston2009_proceedings.htm - proceeding$_0 7 Published Dec 18, 2009. Updated Nov 6, 2015. Accessed Sept 15, 2016.$

[9]DiMaggio C, Ayoung-Chee P, Shinseki M, et al. Traumatic injury in the United States: In-patient epidemiology 2000-2011. Injury. 2016; 47(7):1393-1403.

[10]Barell V, Aharonson-Daniel L, Fingerhut LA, et al. An introduction to the Barell body region by nature of injury diagnosis matrix. Inj Prev, 2002; 8(2): 91-6; Fingerhut L, Aharonson-Daniel L, Mackenzie E, et al. The Barell matrix. Inj Prev, 2002; 8(3): 259.

and means were estimated on the full data set using the R package "sqlsurvey"[11]. Ratio estimates and differences were calculated using simulations based on survey-adjusted counts and standard errors with each simulation consisting of 1,000 random normal draws. Differences in trends over time were evaluated by comparing the slopes of linear regression equations with year as the predictor variable.

We assessed the interaction between pedestrian injury and traumatic brain injury (TBI) on the risk of fatality two ways. First, we tested for the presence of *statistical* interaction in a logistic regression equation with emergency department or post-admission death as the outcome and variables to control for confounding by age and gender, injury severity and treatment at a level 1 or 2 trauma center:

$$death = \beta_{intercept} + \beta_{age} + \beta_{gender} + \beta_{severity} + \beta_{traumaCenter} + \beta_{pedestrian} + \beta_{TBI} + \beta_{pedestrian \cdot TBI}$$

Where, "age" is measured continuously in years, "gender" is an indicator variable for male (0) vs. female (1), "severity" is a dichotmous variable coded 1 for ICISS 0.94, "traumaCenter" is a dichotmous variable coded 1 for treatmetn at a level 1 or 2 trauma center, "pedestrian" is an indicator variable for pedestrian (1) vs cyclist (0) traumatic injury, and TBI is an indicator variable for a Barell matrix designation for traumatic brain injury as the primary diagnostic code. The interaction term, $injury \cdot substance$ indicates the presence of both an ecode for pedestrian injury and a primary diagnostic code for TBI.

We also assessed clinically relevant interaction of pedestrian injury and TBI for fatality on an *additive* scale using an approach described by Darroch (1997) and Rothman and Greenland (1998) within the framework of component causes, where

- $R_{pedestrian \cdot TBI \cdot unknown}$ - the risk of fatality when pedestrian injury and TBI were both present
- $R_{pedestrian \cdot unknown}$ - the risk of fatality when pedestrian injury but not TBI was present
- $R_{TBI \cdot unknown}$ - the risk of fatality when TBI but not pedestrian injury was present
- $R_{unknown}$ - the "background" experience when fatality occurs in the absence of either pedestrian injury or TBI

To determine if the observed $R_{pedestrian \cdot TBI \cdot unknown}$ exceeds what we might expect if the two risks did not interact, we subtract out $R_{pedestrian \cdot unknown}$ and $R_{TBI \cdot unknown}$ and then add back $R_u nknown$ which we subtracted twice, setting up an equality to test the independence of causes:

$$R_{pedestrian \cdot TBI \cdot unknown} = R_{pedestrian \cdot unknown} + R_{TBI \cdot unknown} - R_u nknown$$

Any excess risk beyond these inequalities is considered due to interaction. We assessed interaction on an unadjusted additive scale, in terms of absolute risk differences:

$$(Risk_{pedestrian \cdot substance} - Risk_u nknown) = (Risk_{pedestrian} - Risk_{unknown}) + (Risk_{TBI} - R_{unknown})$$

[11]Thomas Lumley (2014). sqlsurvey: analysis of very large complex survey samples (experimental). R package version 0.6-11/r41. https://R-Forge.R-project.org/projects/sqlsurvey/

The proportion of any excess risk attributable to the interaction between pedestrian injury and TBI was calculated as the excess risk beyond the equality defined in the above equation divided by the overall risk when both pedestrian injury and TBI are present:

$(L_{side} - R_{side})/Risk_{pedestrian \cdot substance}$

We took into account survey variation by running simulation or bootstrap estimates of this equation using point estimates and standard errors returned by survey procedures and report result as a point estimate with 95% confidence intervals.

3 Descriptive Epidemiology

There were 217,551,676 (28396) total emergency department discharges for children under the age of 20 during the study period. A total of 467,093 (se = 1522) of these discharges (0.21% se= 0.0007) were for motor-vehicle related pedestrian or cyclist injuries. Of the total pediatric pedestrian and cyclist discharges, 72.7% (se = 0.4) were pedestrians. There was a 15.1% (se=1.1) decline in the population-based rate of total motor-vehicle related pedestrian and bicycle injury emergency department injuries between 2006 to 2012. The pedestrian injury discharge rate declined 19.3% (se = 1.3). By contrast there was little or no change in the population-based rate of emergency department discharges for motor-vehicle realted cylist injuries, which was 3.2% (se = 2.3) lower in 2012 than in 2006. (Fig **??**fig:injXyr.jpg fig:injXyr.jpgclines for population-based pedestrian and cyclist injury emergency department discharge rates were most notable for 5-to-9 year olds and 10-to-14 year olds, with a shallower decline for those under age 5 and little or no change in rates for 15 to 19 year olds. (Fig **??**fig:injXyrXage.jpg fig:injXyrXage.jpgween the period of 2006 to 2010, population-based pediatric pedestrian and bicyclist injury discharge rates declined in the Midwest and West regions of the United States while remaining essentially unchanged in the Northeast and South. (Fig **??**fig:injXyrXregion fig:injXyrXregion mean age for an injured pediatric pedestrian or cyclist discharged from a US emergency department was 12.2 (se= 0.01) years old. The mean age was 11.9 (se=0.02) for pedestrians and 13.3 (se=0.03) for cyclists. These mean ages did not change appreciably over the study period. Females represented 40.0% (se=0.2) of pediatric pedestrians discharged from US emergency departments and 19.6% (se=0.3) of bicyclists.

```
 # total ped and cyclist under age 20
 (totPedCyclCount<-svytotal(~count, subset(monInj, age < 20 & ped_cycl_mvc==1), se=T,
 na.rm=T, multicore=T))
#                      SE
# count 467093.1 1522.443


 mean(rnorm(1000, mean=467093.1, sd=1522.443)/rnorm(1000, mean=217551676, sd=28396.26))*100
 sd(rnorm(1000, mean=467093.1, sd=1522.443)/rnorm(1000, mean=217551676, sd=28396.26))*100

 # pedestrians
```

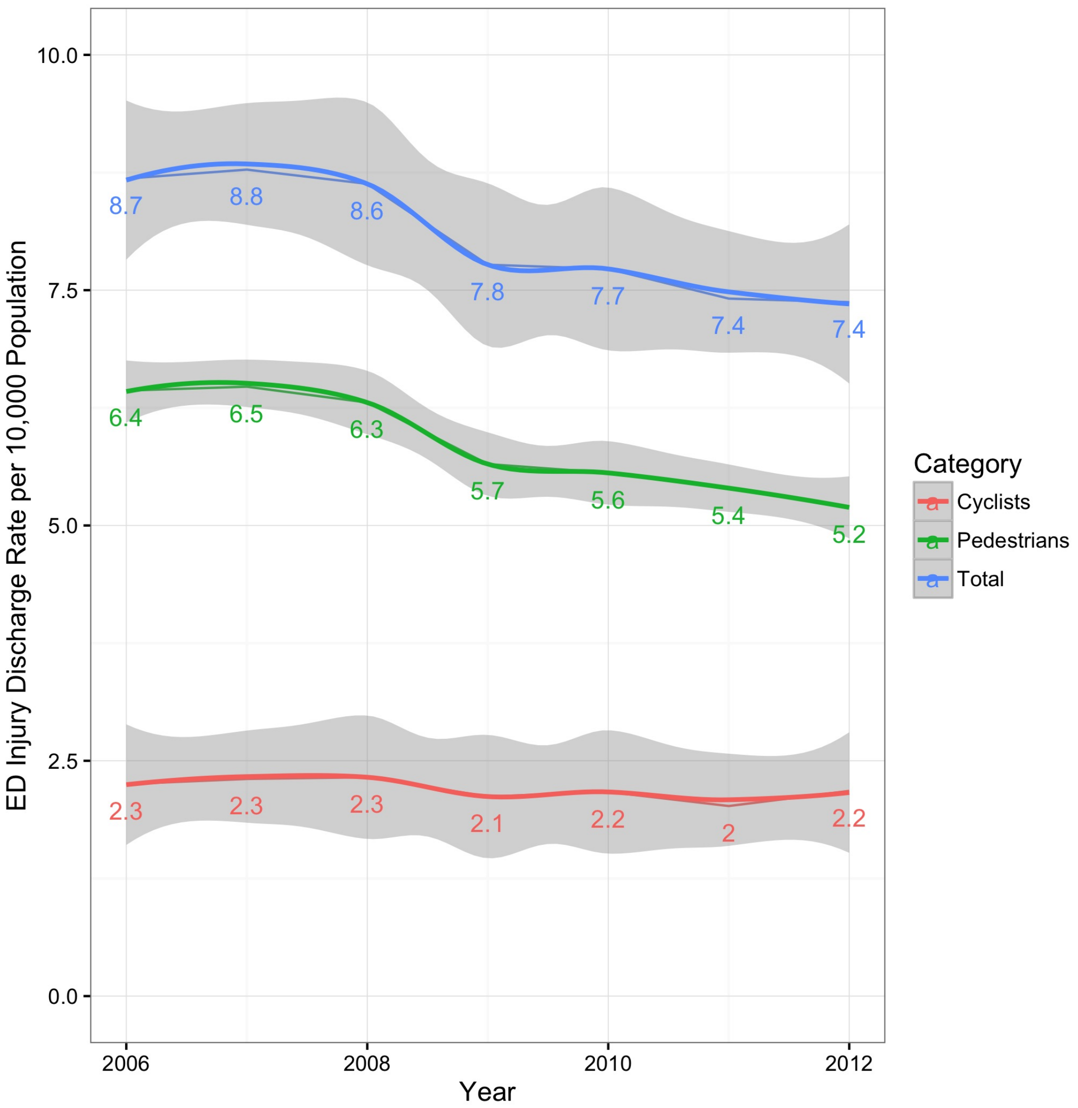

Figure 1: Pediatric Pedestrian and Bicyclist Injury Discharge Rates per 10,000 Population Age 0-19, US Emergency Departments 2006-2012

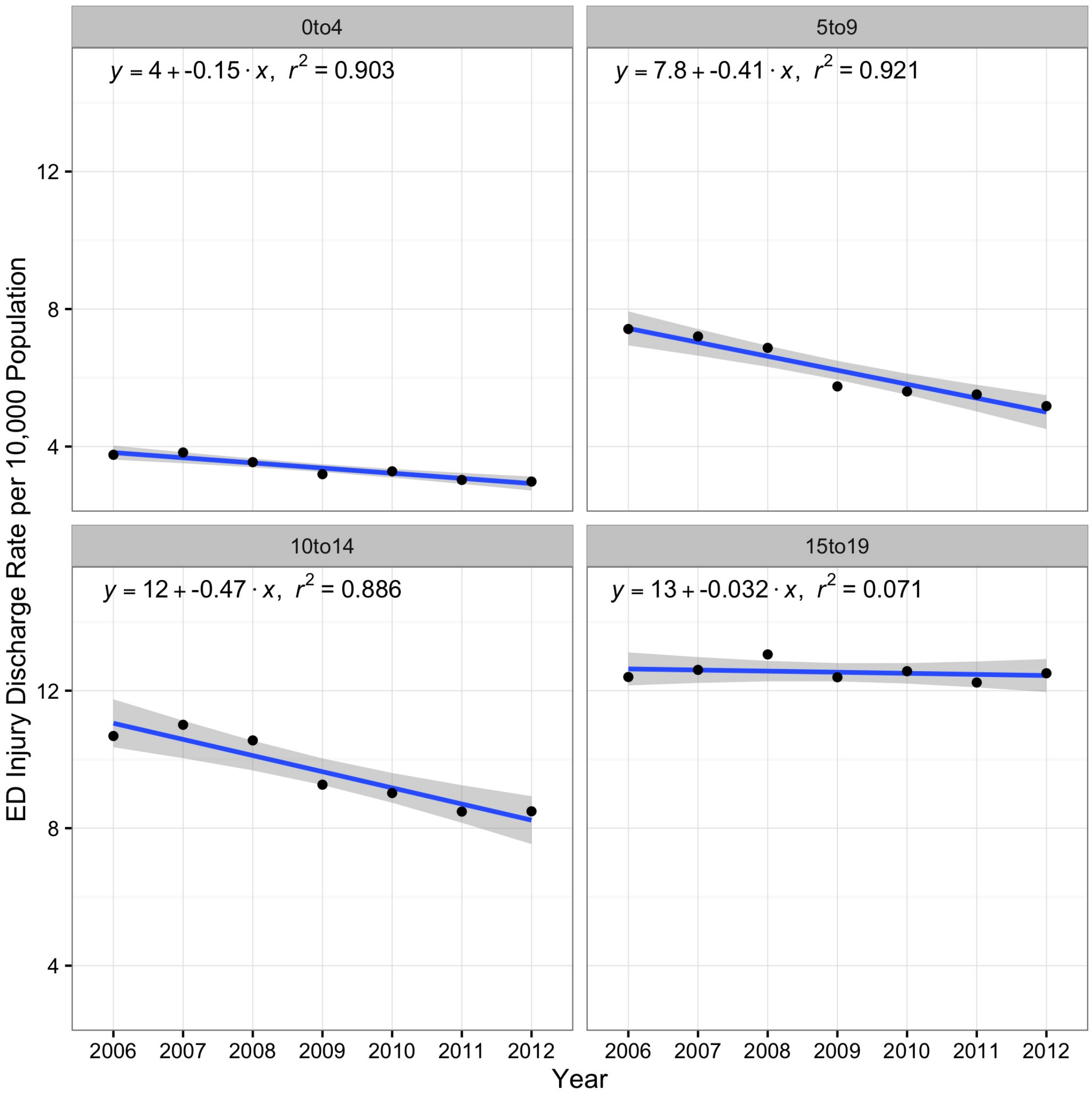

Figure 2: Age-Group Specific Pediatric Total Pedestrian and Bicyclist Injury Discharge Rates per 10,000 Population, US Emergency Departments 2006-2012

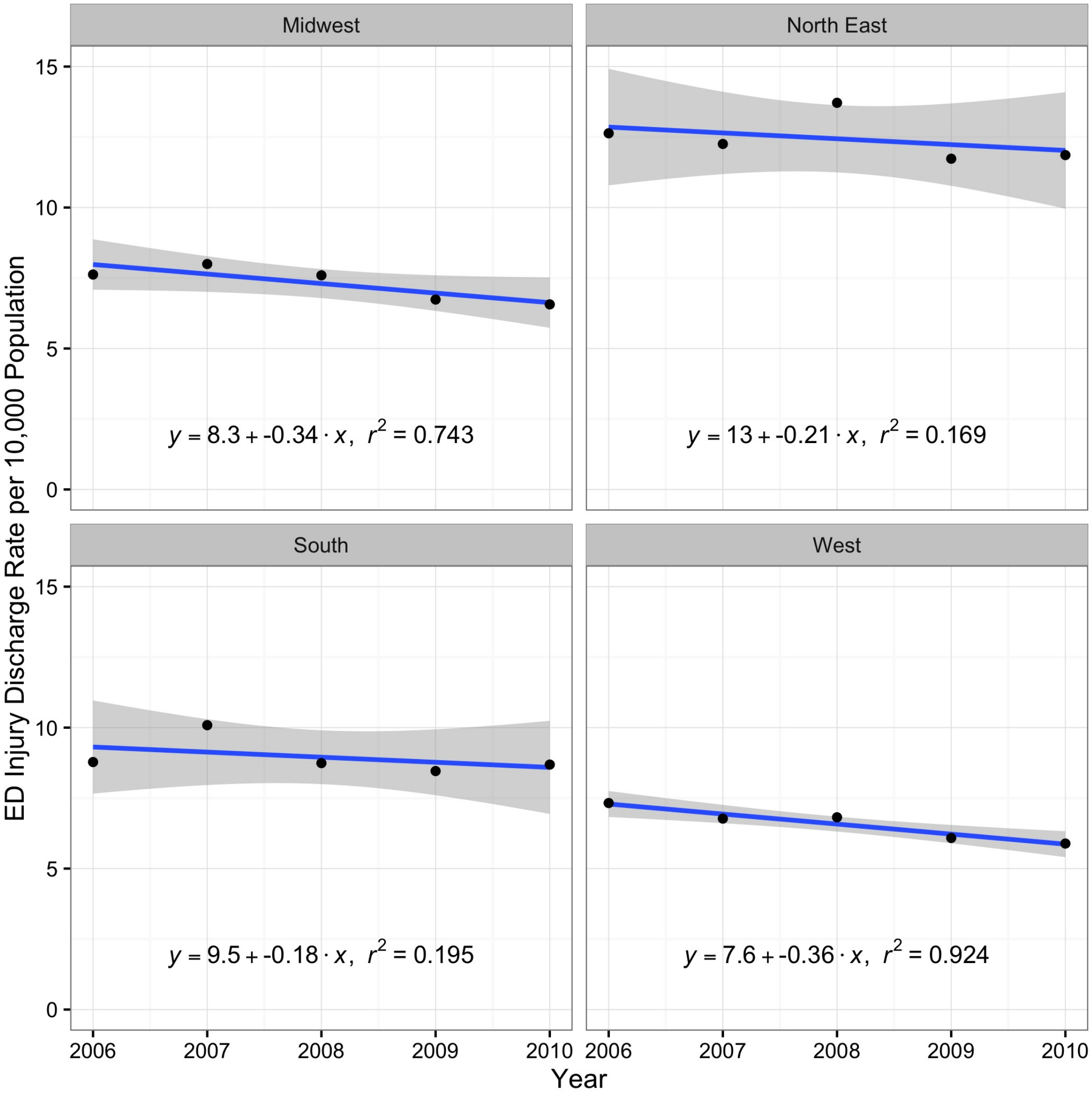

Figure 3: Pediatric Pedestrian and Bicyclist Injury Discharge Rates per 10,000 Population Age 0-19, by Geographic Region, US Emergency Departments 2006-2010

```
(totPedCyclCount<-svytotal(~count, subset(monInj, age < 20 & ped_mvc==1),
se=T, na.rm=T, multicore=T))
#                   SE
# count 339783 1298.37


mean(rnorm(1000, 339783, 1298.37)/rnorm(1000, 467093.1, 1522.443))*100 # 72.74799
sd(rnorm(1000, 339783, 1298.37)/rnorm(1000, 467093.1, 1522.443))*100 # 0.3659932

# cyclists
(totPedCyclCount<-svytotal(~count, subset(monInj, age < 20 & cycl_mvc==1),
se=T, na.rm=T, multicore=T))
#                      SE
# count 127379.4 795.6544


# TOTAL YEARLY NUMBERS

# peds and cyclists
(totPedCyclCountYr<-svytotal(~count, subset(monInj, age < 20 & ped_cycl_mvc==1),
byvar=~yr, se=T, na.rm=T, multicore=T))
#                           SE
# count_2006 71501.24 606.2437
# count_2007 72661.04 599.9264
# count_2008 71728.75 588.5382
# count_2009 64714.18 572.4010
# count_2010 64306.51 572.3481
# count_2011 61370.59 544.0357
# count_2012 60810.76 542.2433


# create age-specific population dataframe (from kidNEDS1.Rnw)
pops<-data.frame(pops=c(rev(c(19989696,20122198,20200529,20244518,20271127,
20125962,19938883)),
rev(c(20470521,20333805,20382409,20182499,19929602,19714611,19544688)),
rev(c(20668585,20711562,20694011,20660564,20706655,20841042,21033138)),
rev(c(21353686,21647328,21959087,22192810,22210880,22067816,21807709))),
ageGroups=c(rep("0to4",7),rep("5to9",7),rep("10to14",7),rep("15to19",7)),yrs=rep(c("2006","2007",
"2008","2009","2010","2011","2012"),4))

plot total injuries by year
tot.rates<-data.frame(Rate=c(71501.24, 72661.04, 71728.75, 64714.18, 64306.51, 61370.59, 60810.76)/
tapply(pops$pops, pops$yrs, sum)*10000, Year=2006:2012)
p1<-ggplot(data=tot.rates, aes(x=Year, y=Rate))
p1+geom_line(aes(group=1))+ylim(0,10)+geom_smooth(aes(group=1))
```

```
tot.rates
#          Rate Year
# 2006 8.685302 2006
# 2007 8.780851 2007
# 2008 8.629722 2008
# 2009 7.770638 2009
# 2010 7.725802 2010
# 2011 7.410574 2011
# 2012 7.372566 2012

(8.685302-7.372566)/8.685302*100 # 15.11445


sim.mean.diff(71501.24/82324418, 606.2437/82324418,60810.76/82482488, 542.2433/82482488) # 15.13858
sim.sd.diff(71501.24/82324418, 606.2437/82324418,60810.76/82482488, 542.2433/82482488) # 1.138372


# populations similar at two time points, simple counts return very similar estimates
sim.mean.diff(71501.24, 606.2437,60810.76, 542.2433) # 14.93836
sim.sd.diff(71501.24, 606.2437,60810.76, 542.2433) # 1.1603




 # peds
 (totPedCyclCountYr<-svytotal(~count, subset(monInj, age < 20 & ped_mvc==1), byvar=~yr,
 se=T, na.rm=T, multicore=T))
#                            SE
# count_2006 52960.76 523.3110
# count_2007 53577.63 516.1629
# count_2008 52416.99 502.8069
# count_2009 47073.64 487.1670
# count_2010 46267.61 485.3252
# count_2011 44646.93 464.6730
# count_2012 42839.48 452.2582


 # plot pedestrian injuries by year
 ped.rates<-data.frame(Rate=c(52960.76, 53577.63, 52416.99, 47073.64, 46267.61, 44646.93, 42839.48)/
 tapply(pops$pops, pops$yrs, sum)*10000, Year=2006:2012)
 p1<-ggplot(data=ped.rates, aes(x=Year, y=Rate))
 p1+geom_line(aes(group=1))+ylim(0,10)+geom_smooth(aes(group=1))

 # decline
 sim.mean.diff(52960.76/82324418, 523.3110/82324418, 42839.48/82482488, 452.2582/82482488) # 19.2605
 sim.sd.diff(52960.76/82324418, 523.3110/82324418, 42839.48/82482488, 452.2582/82482488) # 1.294932
```

```
 # cyclists
 (totPedCyclCountYr<-svytotal(~count, subset(monInj, age < 20 & cycl_mvc==1), byvar=~yr,
 se=T, na.rm=T, multicore=T))
#                            SE
# count_2006 18546.89 306.1665
# count_2007 19092.73 305.8651
# count_2008 19325.85 306.0270
# count_2009 17653.73 300.6462
# count_2010 18054.73 303.5957
# count_2011 16723.66 282.9763
# count_2012 17981.78 299.2923


 # plot cyclist injuries by year
 cyclist.rates<-data.frame(Rate=c(18546.89, 19092.73, 19325.85, 17653.73, 18054.73, 16723.66, 17981.7
 tapply(pops$pops, pops$yrs, sum)*10000, Year=2006:2012)
 p1<-ggplot(data=cyclist.rates, aes(x=Year, y=Rate))
 p1+geom_line(aes(group=1))+ylim(0,5)+geom_smooth(aes(group=1))


 # decline
sim.mean.diff(18546.89/82324418, 306.1665/82324418, 17981.78/82482488, 299.2923/82482488) # 3.181979
sim.sd.diff(18546.89/82324418, 306.1665/82324418, 17981.78/82482488, 299.2923/82482488) # 2.300389

all.rates
#            Rate Year    Category
# 2006  8.685302 2006       Total
# 2007  8.780851 2007       Total
# 2008  8.629722 2008       Total
# 2009  7.770638 2009       Total
# 2010  7.725802 2010       Total
# 2011  7.410574 2011       Total
# 2012  7.372566 2012       Total
# 20061 6.433178 2006 Pedestrians
# 20071 6.474683 2007 Pedestrians
# 20081 6.306314 2008 Pedestrians
# 20091 5.652428 2009 Pedestrians
# 20101 5.558603 2010 Pedestrians
# 20111 5.391172 2011 Pedestrians
# 20121 5.193767 2012 Pedestrians
# 20062 2.252903 2006    Cyclists
# 20072 2.307294 2007    Cyclists
# 20082 2.325103 2008    Cyclists
# 20092 2.119794 2009    Cyclists
# 20102 2.169100 2010    Cyclists
# 20112 2.019402 2011    Cyclists
```

```
# 20122 2.180072 2012    Cyclists
```

```
#combine and plot yearly data for both pedestrians and cyclists

all.rates<-rbind(tot.rates, ped.rates, cyclist.rates)
all.rates$Category<-c(rep("Total",7),rep("Pedestrians",7),rep("Cyclists",7))
all.rates
#            Rate Year    Category
# 2006  8.685302 2006       Total
# 2007  8.780851 2007       Total
# 2008  8.629722 2008       Total
# 2009  7.770638 2009       Total
# 2010  7.725802 2010       Total
# 2011  7.410574 2011       Total
# 2012  7.372566 2012       Total
# 20061 6.433178 2006 Pedestrians
# 20071 6.474683 2007 Pedestrians
# 20081 6.306314 2008 Pedestrians
# 20091 5.652428 2009 Pedestrians
# 20101 5.558603 2010 Pedestrians
# 20111 5.391172 2011 Pedestrians
# 20121 5.193767 2012 Pedestrians
# 20062 2.252903 2006    Cyclists
# 20072 2.307294 2007    Cyclists
# 20082 2.325103 2008    Cyclists
# 20092 2.119794 2009    Cyclists
# 20102 2.169100 2010    Cyclists
# 20112 2.019402 2011    Cyclists
# 20122 2.180072 2012    Cyclists



p1<-ggplot(data=all.rates, aes(x=Year, y=Rate, color=Category))
p2<-p1+geom_line()+geom_smooth()+ylim(0,10)
p3<-p2+ylab("ED Injury Discharge Rate per 10,000 Population")+xlab("Year")+theme_bw()
injXyr<- p3+geom_text(aes(label=round(Rate,1)), vjust=2)

ggsave(file= "~/injXyr.jpg",injXyr)



#  YEARLY NUMERATORS BY AGE GROUP

# # PEDS AND CYCLISTS
```

```
# younger than 5
(yrCountPedCyclAge1<-svytotal(~count,subset(monInj, agecat_kids==1 & ped_cycl_mvc==1),
byvar=~yr, se=T, na.rm=T, multicore=T))
                          SE
count_2006 7496.920 197.8304
count_2007 7701.300 193.5445
count_2008 7187.447 186.7201
count_2009 6469.181 183.4573
count_2010 6622.841 182.7178
count_2011 6092.855 171.5372
count_2012 5958.418 170.1881

# 5 to 9
(yrCountPedCyclAge2<-svytotal(~count,subset(monInj, agecat_kids==2 & ped_cycl_mvc==1),
byvar=~yr, se=T, na.rm=T, multicore=T))
                          SE
count_2006 14499.86 275.6748
count_2007 14201.23 264.3805
count_2008 13692.70 258.7592
count_2009 11603.26 243.5144
count_2010 11418.74 240.4029
count_2011 11215.76 233.6923
count_2012 10597.25 225.6384

# 10 to 14
(yrCountPedCyclAge3<-svytotal(~count,subset(monInj, agecat_kids==3 & ped_cycl_mvc==1),
byvar=~yr, se=T, na.rm=T, multicore=T))
                          SE
count_2006 22467.23 342.2771
count_2007 22940.62 336.7747
count_2008 21851.66 323.6377
count_2009 19142.44 311.4260
count_2010 18670.09 307.1942
count_2011 17569.73 291.0733
count_2012 17548.18 291.2927

# 15 to 19
(yrCountPedCyclAge4<-svytotal(~count,subset(monInj, agecat_kids==4 & ped_cycl_mvc==1),
byvar=~yr, se=T, na.rm=T, multicore=T))
                          SE
count_2006 27037.24 368.6608
count_2007 27817.89 373.8933
count_2008 28996.95 374.7876
count_2009 27499.30 371.8471
count_2010 27594.84 377.6729
count_2011 26492.24 357.4273
```

```
count_2012 26706.91 360.3822


 age.rates<-pops
 age.rates$Count<- c(7496.920, 7701.300, 7187.447, 6469.181, 6622.841, 6092.855, 5958.418,
 14499.86, 14201.23, 13692.70, 11603.26, 11418.74, 11215.76, 10597.25, 22467.23, 22940.62,
 21851.66, 19142.44, 18670.09, 17569.73, 17548.18, 27037.24, 27817.89, 28996.95, 27499.30, 27594.84,
 age.rates$Rate<-age.rates$Count/age.rates$pop*10000
 age.rates$ageGroups_f<-factor(age.rates$ageGroups,
 levels = c("0to4","5to9","10to14","15to19")) # factor version of age groups to arrange properly on fa
 age.rates
#          pops ageGroups  yrs      Count      Rate ageGroups_f
# 1  19938883      0to4 2006  7496.920  3.759950        0to4
# 2  20125962      0to4 2007  7701.300  3.826550        0to4
# 3  20271127      0to4 2008  7187.447  3.545657        0to4
# 4  20244518      0to4 2009  6469.181  3.195522        0to4
# 5  20200529      0to4 2010  6622.841  3.278548        0to4
# 6  20122198      0to4 2011  6092.855  3.027927        0to4
# 7  19989696      0to4 2012  5958.418  2.980745        0to4
# 8  19544688      5to9 2006 14499.860  7.418824        5to9
# 9  19714611      5to9 2007 14201.230  7.203404        5to9
# 10 19929602      5to9 2008 13692.700  6.870534        5to9
# 11 20182499      5to9 2009 11603.260  5.749169        5to9
# 12 20382409      5to9 2010 11418.740  5.602252        5to9
# 13 20333805      5to9 2011 11215.760  5.515820        5to9
# 14 20470521      5to9 2012 10597.250  5.176835        5to9
# 15 21033138    10to14 2006 22467.230 10.681825      10to14
# 16 20841042    10to14 2007 22940.620 11.007425      10to14
# 17 20706655    10to14 2008 21851.660 10.552965      10to14
# 18 20660564    10to14 2009 19142.440  9.265207      10to14
# 19 20694011    10to14 2010 18670.090  9.021977      10to14
# 20 20711562    10to14 2011 17569.730  8.483054      10to14
# 21 20668585    10to14 2012 17548.180  8.490267      10to14
# 22 21807709    15to19 2006 27037.240 12.398019      15to19
# 23 22067816    15to19 2007 27817.890 12.605638      15to19
# 24 22210880    15to19 2008 28996.950 13.055291      15to19
# 25 22192810    15to19 2009 27499.300 12.391085      15to19
# 26 21959087    15to19 2010 27594.840 12.566479      15to19
# 27 21647328    15to19 2011 26492.240 12.238111      15to19
# 28 21353686    15to19 2012 26706.910 12.506932      15to19


 # # function to add regression equation to ggplot

 lm_eqn <- function(df){
    m <- lm(Rate ~ as.numeric(yrs), df); # note yrs is factor, need to coerce to numeric
     eq <- substitute(italic(y) == a + b %.% italic(x)*","~~italic(r)^2~"="~r2,
```

```
          list(a = format(coef(m)[1], digits = 2),
               b = format(coef(m)[2], digits = 2),
              r2 = format(summary(m)$r.squared, digits = 3)))
     as.character(as.expression(eq));
}


# # working with grid_wrap, have to create equation labels for the groups

eq <- ddply(age.rates,.(ageGroups_f),lm_eqn)

p1<-ggplot(data=age.rates, aes(x=yrs, y=Rate))
(p2<-p1+geom_smooth(method="lm", aes(group=1)) + geom_point() + facet_wrap(~ageGroups_f))
(p3<-p2+ geom_text(data=eq,aes(x = 3, y = 15,label=V1), parse = TRUE, inherit.aes=FALSE))
(injXyrXage<-p3+ylab("ED Injury Discharge Rate per 10,000 Population")+xlab("Year")+theme_bw())
ggsave(file="~/injXyrXage.jpg",injXyrXage)


# BREAK OUT AGE-BASED PED AND CYCLIST NUMBERS

# PEDS
# younger than 5
(yrCountPedCyclAge1<-svytotal(~count,subset(monInj, agecat_kids==1 & ped_mvc==1),
byvar=~yr, se=T, na.rm=T, multicore=T))
                          SE
count_2006 7111.476 192.9798
count_2007 7318.379 188.2899
count_2008 6733.847 180.4918
count_2009 6126.605 178.0055
count_2010 6234.805 176.8340
count_2011 5714.393 166.0917
count_2012 5551.893 164.4773

# 5 to 9
(yrCountPedCyclAge2<-svytotal(~count,subset(monInj, agecat_kids==2 & ped_mvc==1),
 byvar=~yr, se=T, na.rm=T, multicore=T))
                           SE
count_2006 10919.716 240.0338
count_2007 10694.022 230.1401
count_2008 10440.512 226.2904
count_2009  8833.370 211.5374
count_2010  8518.909 205.5400
count_2011  8494.419 203.0655
count_2012  8074.755 196.6735

# 10 to 14
(yrCountPedCyclAge3<-svytotal(~count,subset(monInj, agecat_kids==3 & ped_mvc==1),
```

```
 byvar=~yr, se=T, na.rm=T, multicore=T))
                          SE
count_2006 14769.23 279.0869
count_2007 15002.57 272.5823
count_2008 14156.18 260.1270
count_2009 12325.54 248.5436
count_2010 12108.57 247.2242
count_2011 11854.37 238.9647
count_2012 11148.26 230.2350

 # 15 to 19
 (yrCountPedCyclAge4<-svytotal(~count,subset(monInj, agecat_kids==4 & ped_mvc==1),
  byvar=~yr, se=T, na.rm=T, multicore=T))
                          SE
count_2006 20160.34 318.5951
count_2007 20562.67 322.6229
count_2008 21086.45 318.9585
count_2009 19788.12 315.3509
count_2010 19405.32 318.1825
count_2011 18583.75 300.4935
count_2012 18064.58 293.4224

 # CYCLISTS
  # CYCLISTS

 # younger than 5
 (yrCountPedCyclAge1<-svytotal(~count,subset(monInj, agecat_kids==1 & cycl_mvc==1),
 byvar=~yr, se=T, na.rm=T, multicore=T))
                          SE
count_2006 385.4440 43.33510
count_2007 382.9209 44.60150
count_2008 453.6004 47.64858
count_2009 342.5768 44.22297
count_2010 388.0366 45.83163
count_2011 378.4615 42.71921
count_2012 406.5246 43.54047

 # 5 to 9
 (yrCountPedCyclAge2<-svytotal(~count,subset(monInj, agecat_kids==2 & cycl_mvc==1),
 byvar=~yr, se=T, na.rm=T, multicore=T))
                          SE
count_2006 3580.139 135.4131
count_2007 3507.203 129.9614
count_2008 3252.189 125.3356
count_2009 2774.586 120.5852
count_2010 2899.826 124.5643
count_2011 2721.342 115.5212
```

```
count_2012 2527.214 110.5593

 # 10 to 14
 (yrCountPedCyclAge3<-svytotal(~count,subset(monInj, agecat_kids==3 & cycl_mvc==1),
  byvar=~yr, se=T, na.rm=T, multicore=T))
                           SE
count_2006 7698.002 197.9648
count_2007 7942.734 197.6396
count_2008 7709.567 192.5336
count_2009 6820.703 187.5169
count_2010 6577.345 182.4194
count_2011 5715.364 166.0267
count_2012 6399.928 178.2757

 # 15 to 19
 (yrCountPedCyclAge4<-svytotal(~count,subset(monInj, agecat_kids==4 & cycl_mvc==1),
  byvar=~yr, se=T, na.rm=T, multicore=T))
                           SE
count_2006 6883.302 185.3897
count_2007 7259.870 188.8151
count_2008 7910.493 196.5796
count_2009 7715.869 196.8867
count_2010 8189.518 203.2626
count_2011 7908.494 193.3338
count_2012 8648.115 209.0977




 # COUNTS BY REGION (NB: ONLY 5-YRS DATA, 2006-2010)




  (totPedCyclCountYr<-svytotal(~ped_cycl_mvc, subset(monInj, age < 20),
  byvar=~region, se=T, na.rm=T, multicore=T))
#                              SE
# ped_cycl_mvc0_1 28714481.30 NA
# ped_cycl_mvc1_1    87586.09 NA
# ped_cycl_mvc0_2 36434743.13 NA
# ped_cycl_mvc1_2    66150.58 NA
# ped_cycl_mvc0_3 59227228.20 NA
# ped_cycl_mvc1_3   101468.42 NA
# ped_cycl_mvc0_4 29924798.59 NA
# ped_cycl_mvc1_4    89706.62 NA
```

```r
  (totPedCyclCountYr<-svytotal(~count, subset(monInj, age < 20 & ped_cycl_mvc==1),
  byvar=~region, se=T, na.rm=T, multicore=T))
#                    SE
# count_1  87586.09 NA
# count_2  66150.58 NA
# count_3 101468.42 NA
# count_4  89706.62 NA

  # YEARLY COUNTS BY REGION

  (totPedCyclCountYr<-svytotal(~ped_cycl_mvc, subset(monInj, age < 20 & region==1),
  byvar=~yr, se=T, na.rm=T, multicore=T))
#                                     SE
# ped_cycl_mvc0_2006 5509716.42 5163.9172
# ped_cycl_mvc1_2006   17982.01  312.8603
# ped_cycl_mvc0_2007 5634376.93 5386.0719
# ped_cycl_mvc1_2007   17289.67  302.3081
# ped_cycl_mvc0_2008 6008636.84 5292.6327
# ped_cycl_mvc1_2008   19360.05  313.1879
# ped_cycl_mvc0_2009 6154417.19 5288.2571
# ped_cycl_mvc1_2009   16472.89  280.9903
# ped_cycl_mvc0_2010 5407333.92 4895.0521
# ped_cycl_mvc1_2010   16481.46  279.9101



reg1<-c(17982.01,17289.67,19360.05,16472.89,16481.46)


  (totPedCyclCountYr<-svytotal(~ped_cycl_mvc, subset(monInj, age < 20 & region==2),
  byvar=~yr, se=T, na.rm=T, multicore=T))
#                                     SE
# ped_cycl_mvc0_2006 7342013.72 6020.6549
# ped_cycl_mvc1_2006   13831.92  271.5383
# ped_cycl_mvc0_2007 7310361.73 6199.2657
# ped_cycl_mvc1_2007   14494.08  287.0758
# ped_cycl_mvc0_2008 7338186.01 6013.2371
# ped_cycl_mvc1_2008   13751.40  266.5978
# ped_cycl_mvc0_2009 7700880.66 6285.6449
# ped_cycl_mvc1_2009   12202.00  257.9999
# ped_cycl_mvc0_2010 6743301.01 6079.2657
# ped_cycl_mvc1_2010   11871.18  258.7533



 reg2 <- c(13831.92,14494.08,13751.40,12202.00,11871.18)
```

```r
 (totPedCyclCountYr<-svytotal(~ped_cycl_mvc, subset(monInj, age < 20 & region==3),
  byvar=~yr, se=T, na.rm=T, multicore=T))
#                                        SE
# ped_cycl_mvc0_2006 11681708.53 7374.1996
# ped_cycl_mvc1_2006    22214.16  330.9561
# ped_cycl_mvc0_2007 11740084.90 7268.6461
# ped_cycl_mvc1_2007    20797.41  320.2876
# ped_cycl_mvc0_2008 11353616.51 6968.6565
# ped_cycl_mvc1_2008    21027.69  315.8852
# ped_cycl_mvc0_2009 12692542.55 7815.2690
# ped_cycl_mvc1_2009    18965.88  315.2366
# ped_cycl_mvc0_2010 11759275.70 7298.4394
# ped_cycl_mvc1_2010    18463.27  302.3754



reg3 <- c(22214.16,20797.41,21027.69,18965.88,18463.27)


 (totPedCyclCountYr<-svytotal(~ped_cycl_mvc, subset(monInj, age < 20 & region==4),
 byvar=~yr, se=T, na.rm=T, multicore=T))
#                                       SE
# ped_cycl_mvc0_2006 5821196.03 5816.9166
# ped_cycl_mvc1_2006   17473.15  320.2159
# ped_cycl_mvc0_2007 6305002.79 5745.6842
# ped_cycl_mvc1_2007   20079.87  316.6446
# ped_cycl_mvc0_2008 5879519.94 5499.6137
# ped_cycl_mvc1_2008   17589.61  304.6928
# ped_cycl_mvc0_2009 6151310.61 5790.2893
# ped_cycl_mvc1_2009   17073.40  312.8998
# ped_cycl_mvc0_2010 5767769.22 5488.3269
# ped_cycl_mvc1_2010   17490.60  320.8629



reg4 <- c(17473.15,20079.87,17589.61,17073.40,17490.60)

# PLOT COUNTS BY REGION

# populations (see pedPopsRegion.xls)
NE1 <- c(14232751.78,14107601.51, 14115668.2, 14042054.72 , 13895620.04)
MW2 <- c(18143659.66,18124141.58, 18104714.94 , 18112804.24 , 18083478.96)
W3 <- c(30325283.33,30706430.51, 30834319.52 , 31162416.73 , 31358342.65)
S4 <- c(19905069.54,19907533.8 , 20122805.23 , 20182198.84 , 20129035.6 )

counts<-c(reg1, reg2, reg3, reg4)
```

```
region.dat<-data.frame(Year=rep(2006:2010,4), Region=c(rep("North East",5),
rep("Midwest",5),rep("West",5),rep("South",5)),Population=c(NE1, MW2,W3,S4), Count=counts )
region.dat$Rate<-region.dat$Count/region.dat$Population*10000
region.dat$yrs<-region.dat$Year-2005
region.dat


eq <- ddply(region.dat,.(Region),lm_eqn) # see def of lm_eqn above

p1<-ggplot(data=region.dat, aes(x=Year, y=Rate))
(p2<-p1+geom_smooth(method="lm", aes(group=1)) + geom_point() +ylim(0,15)
 + facet_wrap(~Region))
(p3<-p2+ geom_text(data=eq,aes(x = 2008, y = 2,label=V1), parse = TRUE,
inherit.aes=FALSE))

(injXyrXregion<-p3+ylab("ED Injury Discharge Rate per 10,000 Population")
+xlab("Year")+theme_bw())

ggsave(file="~/injXyrXregion.jpg",injXyrXregion)




# AGE
# peds and cyclists
 (svymean(~age, subset(monInj, age < 20 & ped_cycl_mvc==1),
 na.rm=T,multicore=T, se=T))
#                     SE
# age 12.23946 0.01763518


 (svymean(~age, subset(monInj, age < 20 & ped_cycl_mvc==1),
 na.rm=T,multicore=T, se=T, byvar=~yr))
#                          SE
# age_2006 11.92410 0.04559854
# age_2007 11.93473 0.04628597
# age_2008 12.21585 0.04342217
# age_2009 12.40369 0.04567927
# age_2010 12.38125 0.04866329
# age_2011 12.40362 0.04957935
# age_2012 12.51184 0.04737534

# peds
 (svymean(~age, subset(monInj, age < 20 & ped_mvc==1),
 na.rm=T,multicore=T, se=T))
#                     SE
```

```
# age 11.85596 0.02207554

  (svymean(~age, subset(monInj, age < 20 & ped_mvc==1),
  na.rm=T,multicore=T, se=T, byvar=~yr))
#                            SE
# age_2006 11.61046 0.05697846
# age_2007 11.58839 0.05836447
# age_2008 11.86054 0.05418841
# age_2009 12.02494 0.05625212
# age_2010 11.96214 0.06171677
# age_2011 12.00303 0.06144844
# age_2012 12.03488 0.05980054




  (svymean(~age, subset(monInj, age < 20 & cycl_mvc==1),
  na.rm=T,multicore=T, se=T))
#                       SE
# age 13.26278 0.02568442

  (svymean(~age, subset(monInj, age < 20 & cycl_mvc==1),
  na.rm=T,multicore=T, se=T, byvar=~yr))
#                            SE
# age_2006 12.82215 0.06416131
# age_2007 12.90811 0.06256198
# age_2008 13.17971 0.06349926
# age_2009 13.41355 0.07185998
# age_2010 13.45343 0.06843185
# age_2011 13.47308 0.07595545
# age_2012 13.64808 0.06925867


 # GENDER

 svytotal(~female, subset(monInj, age < 20 & ped_cycl_mvc==1),
 se=T, na.rm=T, multicore=T)
#                        SE
# female 160838.5 935.2522

  svymean(~female, subset(monInj, age < 20 & ped_cycl_mvc==1),
  se=T, na.rm=T, multicore=T)
#                            SE
# female 0.3443393 0.001657114


  svymean(~female, subset(monInj, age < 20 & ped_mvc==1), se=T,
```

```
  na.rm=T, multicore=T)
#                           SE
# female 0.3999558 0.002000165

  svymean(~female, subset(monInj, age < 20 & cycl_mvc==1), se=T,
  na.rm=T, multicore=T)
#                           SE
# female 0.1959147 0.002693529
```

While, the total ED-based visit rate of severely injured (ICISS ¡ 0.94) of all pediatric emergency department discharges during the study period was 104.2 (se = 0.7) per 10,000 ED discharges of children under age 19, the rate of severely injured pediatric pedestrians and bicyclists per 10,000 discharges in that age group was 920.0 (se = 22.7), or 8.8 (95% CI 7.9, 9.8) time greater. There was no meaningful difference in the severe injury ED-based discharge rate between pedestrians (946.0 per 10,0000 discharges, se = 27.1) and cyclists (919.5 per 10,000 discharges, se = 60.3) There was little or no change in these rates over the study period.

4 Injury Severity

```
# total proportion severe injuries
svymean(~severe, subset(monInj, age < 20),
na.rm=T,multicore=T, se=T)
#                               SE
# _severe 0.01041865 6.890667e-05
0.01041865*10000 # 104.1865
6.890667e-05 * 10000 # 0.6890667

# total severity peds and cyclists
  svymean(~severe, subset(monInj, age < 20 & ped_cycl_mvc==1),
  na.rm=T,multicore=T, se=T)
#                             SE
# severe 0.09199237 0.002272958
0.09199237 * 10000 # 919.9237
0.002272958 * 10000 # 22.72958




mean(rnorm(1000, 0.09199237, 0.002272958)/
rnorm(1000,0.01041865, 6.890667e-05)) # 8.822565
sd(rnorm(1000, 0.09199237, 0.002272958)/
rnorm(1000,0.01041865, 6.890667e-05)) # 0.2259549
8.822565 - 1.96*sqrt(0.2259549) # 7.890885
8.822565 + 1.96*sqrt(0.2259549) 9.754245
```

```
  # total severity peds
  svymean(~severe, subset(monInj, age < 20 & ped_mvc==1),
  na.rm=T,multicore=T, se=T)
#                             SE
# severe 0.09459487 0.002708105
0.09459487*10000 # 943.3103
0.002708105*10000 # 26.89051

 # total severity cyclists
 svymean(~severe, subset(monInj, age < 20 & cycl_mvc==1),
 na.rm=T,multicore=T, se=T)
#                              SE
# _severe 0.09194759 0.006028156
0.09194759*10000 # 919.4759
0.006028156 * 10000 # 60.28156

 # yearly severity peds and cyclists
 svymean(~severe, subset(monInj, age < 20 & ped_cycl_mvc==1),
 byvar=~yr, na.rm=T,multicore=T, se=T)
#                                 SE
# severe_2006 0.08870085 0.005786656
# severe_2007 0.09334003 0.005669393
# severe_2008 0.09582659 0.005812242
# severe_2009 0.08942927 0.006017442
# severe_2010 0.09540582 0.006299878
# severe_2011 0.08602648 0.006128577
# severe_2012 0.09490409 0.006482906


  # yearly severity peds
  svymean(~severe, subset(monInj, age < 20 & ped_mvc==1),
  byvar=~yr, na.rm=T,multicore=T, se=T)
#                                 SE
# severe_2006 0.09123751 0.006860675
# severe_2007 0.09564254 0.006687330
# severe_2008 0.09726539 0.006836147
# severe_2009 0.09205410 0.007163789
# severe_2010 0.09886052 0.007606822
# severe_2011 0.08803247 0.007318828
# severe_2012 0.09925894 0.007869296


  # yearly severity  cyclists
  svymean(~severe, subset(monInj, age < 20 & cycl_mvc==1),
  byvar=~yr, na.rm=T,multicore=T, se=T)
#                                SE
# severe_2006 0.08144083 0.01066592
```

```
# severe_2007 0.08692906 0.01065687
# severe_2008 0.09189589 0.01102966
# severe_2009 0.08244769 0.01103478
# severe_2010 0.08723263 0.01116856
# severe_2011 0.08079851 0.01115910
# severe_2012 0.08481450 0.01137419
```

Between 2006 and 2012, pedestrians and cyclists accounted for 3,531 (se = 135), ED deaths among persons age 19 or younger, or 3.5% (se=0.1) of the total 101,147 (se=717) ED deaths in that age group. Pedestrians accounted for 3,042 (se=126) or 86.6% (se=5.0) of the total pedestrian and cyclist ED deaths. The unadjusted relative risk estimate for the association of pediatric pedestrian injury with fatality compared to cyclist injury was 2.4 (95% CI 1.8, 2.9). There was a 24.8% (95% CI 17.8, 31.9) decline in the population-based rate of pediatric pedestrian and cyclist ED deaths from 7.3 (se=0.7) deaths per 1,000,000 US population 0 to 19 in 2006 to 5.4 (se=0.6) in 2012. The case fatality for pedestrian or bicyclist ED discharges for the population under age 19 decreased 14.0% (95% CI 4.5, 19.2) from 83.7 (se=8.1) deaths per 10,000 visits in 2006 to 73.7 (se=7.7) deaths per 10,000 visits in 2012.

5 Deaths

```
# total kids ed (pre and post admit) deaths
  svytotal(~died_any, subset(monInj, age < 20),
  na.rm=T,multicore=T, se=T)
#                         SE
# _died_any 101147.3 716.716

 # total deaths peds and cyclists
 svytotal(~died_any, subset(monInj, age < 20 & ped_cycl_mvc==1),
 na.rm=T,multicore=T, se=T)
#                         SE
# _died_any 3530.833 135.4656


# proportion ped cycl deaths among total deaths
 mean(rnorm(1000, 3530.833, 135.4656)/
 rnorm(1000, 101147.3,716.716))*100 # 3.487794
 sd(rnorm(1000, 3530.833, 135.4656)/
 rnorm(1000, 101147.3,716.716))*100 # 0.1414614


 # total deaths peds
 svytotal(~died_any, subset(monInj, age < 20 & ped_mvc==1),
 na.rm=T,multicore=T, se=T)
#                          SE
# _died_any 3042.36 125.5156
```

```
# total deaths cyclists
svytotal(~died_any, subset(monInj, age < 20 & cycl_mvc==1),
na.rm=T,multicore=T, se=T)
#                          SE
# _died_any 488.4732 50.95896

# proportion ped deaths among total ped cycl deaths

mean(rnorm(1000, 3042.36, 125.5156)/
rnorm(1000, 3530.833, 135.4656))*100 # 86.64646
sd(rnorm(1000, 3042.36, 125.5156)/
rnorm(1000, 3530.833, 135.4656))*100 # 5.004179


#proportion bike deaths among total ped cycl deaths
mean(rnorm(1000,488.4732, 50.95896)/
rnorm(1000, 3530.833, 135.4656))*100 # 13.91003
sd(rnorm(1000,488.4732, 50.95896)/
rnorm(1000, 3530.833, 135.4656))*100 # 1.519205

# RR ped deaths vs bike deaths
# (count ped deaths/ total peds)/ (count bike deaths/total bike)

mean((rnorm(1000, 3042.36, 125.5156)/rnorm(1000, 339783, 1298.37))/
(rnorm(1000,488.4732, 50.95896)/rnorm(1000, 127379.4, 795.6544))) # 2.358354
sd((rnorm(1000, 3042.36, 125.5156)/rnorm(1000, 339783, 1298.37))/
(rnorm(1000,488.4732, 50.95896)/rnorm(1000, 127379.4, 795.6544)))# 0.2670858
2.358354 -(1.96*0.2670858) # 1.834866
2.358354 +(1.96*0.2670858) # 2.881842

# yearly deaths peds and cyclists
svytotal(~died_any, subset(monInj, age < 20 & ped_cycl_mvc==1),
byvar=~yr, na.rm=T,multicore=T, se=T)
#                               SE
# _died_any_2006 599.9949 55.70074
# _died_any_2007 535.7119 56.13028
# _died_any_2008 602.6810 55.24830
# _died_any_2009 495.9098 49.63711
# _died_any_2010 467.9819 48.30128
# _died_any_2011 379.9875 44.06526
# _died_any_2012 448.5659 48.03025



 # yearly deaths peds
```

```
svytotal(~died_any, subset(monInj, age < 20 & ped_mvc==1),
byvar=~yr, na.rm=T,multicore=T, se=T)
#                                SE
# _died_any_2006 515.1962 51.54674
# _died_any_2007 463.2789 53.06611
# _died_any_2008 511.1823 51.14444
# _died_any_2009 425.5369 45.38629
# _died_any_2010 409.6427 44.06678
# _died_any_2011 331.8504 41.26890
# _died_any_2012 385.6721 44.27763


 # yearly deaths  cyclists
 svytotal(~died_any, subset(monInj, age < 20 & cycl_mvc==1),
 byvar=~yr, na.rm=T,multicore=T, se=T)
#                                 SE
# _died_any_2006 84.79865 21.10705
# _died_any_2007 72.43293 18.29203
# _died_any_2008 91.49869 20.89551
# _died_any_2009 70.37291 20.09799
# _died_any_2010 58.33924 19.77711
# _died_any_2011 48.13710 15.44749
# _died_any_2012 62.89371 18.61187


# PLOT AND PERCENTAGE POPULATION-BASED DECLINE

# create age-specific population dataframe (from kidNEDS1.Rnw)
pops<-data.frame(pops=c(rev(c(19989696,20122198,20200529,
20244518,20271127,20125962,19938883)),
rev(c(20470521,20333805,20382409,20182499,19929602,
19714611,19544688)),
rev(c(20668585,20711562,20694011,20660564,20706655,
20841042,21033138)),
rev(c(21353686,21647328,21959087,22192810,22210880,
22067816,21807709))),
ageGroups=c(rep("0to4",7),rep("5to9",7),rep("10to14",7),rep("15to19",7)),
yrs=rep(c("2006","2007","2008","2009","2010","2011","2012"),4))

tapply(pops$pops, pops$yrs, sum)
#     2006     2007     2008     2009     2010     2011     2012
# 82324418 82749431 83118264 83280391 83236036 82814893 82482488


# plot total deaths by year
tot.rates<-data.frame(Rate=c(599.9949, 535.7119, 602.6810, 495.9098, 4
67.9819, 379.9875, 448.5659)/tapply(pops$pops, pops$yrs, sum)*1000000, Year=2006:2012)
```

```
p1<-ggplot(data=tot.rates, aes(x=Year, y=Rate))
p1+geom_line(aes(group=1))+ylim(0,15)+geom_smooth(aes(group=1))



# rate 2006
mean(rnorm(1000,599.9949, 55.70074))/82324418*1000000 #7.285612
sd(rnorm(1000,599.9949, 55.70074))/82324418*1000000 # 0.6730783

# rate 2012
mean(rnorm(1000,448.5659, 48.03025))/82482488*1000000 #5.43454
sd(rnorm(1000,448.5659, 48.03025))/82482488*1000000 # 0.6000059


# diff decline  ped and cycl
sim.mean.diff(599.9949, 55.70074, 448.5659, 48.03025) # 24.84779
sim.sd.diff(599.9949, 55.70074, 448.5659, 48.03025) # 12.7913


24.84779-1.96*sqrt(12.7913) # 17.83786
24.84779+1.96*sqrt(12.7913) # 31.85772


# CASE FATALITY



# TOTAL CFR
# total yearly deaths
svytotal(~died_any, subset(monInj, age < 20),byvar=~yr,
na.rm=T,multicore=T, se=T)

#                                 SE
# _died_any_2006 15772.03 289.1264
# _died_any_2007 17303.19 295.6646
# _died_any_2008 15182.13 271.5049
# _died_any_2009 13902.41 264.8936
# _died_any_2010 14148.00 272.9921
# _died_any_2011 13208.74 257.8920
# _died_any_2012 11630.76 240.4615

# total yearly visits
svytotal(~count, subset(monInj, age < 20),byvar=~yr,
na.rm=T,multicore=T, se=T)
#                              SE
# _count_2006 30426136 12098.69
# _count_2007 31062487 12167.47
```

```
# _count_2008 30651688 11765.81
# _count_2009 32763865 12488.62
# _count_2010 29741986 11786.65
# _count_2011 31060570 11708.42
# _count_2012 31844943 11880.94


# total CRF 2006
mean(rnorm(1000,15772.03, 289.1264)/
rnorm(1000,30426136, 12098.69))*10000 # 5.182254
sd(rnorm(1000,15772.03, 289.1264)/
rnorm(1000,30426136, 12098.69))*10000 # 0.0962532


# total CRF 2012
mean(rnorm(1000,11630.76, 240.4615)/
rnorm(1000,31844943, 11880.94))*10000 # 3.645762
sd(rnorm(1000,11630.76, 240.4615)/
rnorm(1000,31844943, 11880.94))*10000 # 0.07677712

sim.mean.diff(5.182254,0.0962532,3.645762,0.07677712) # 29.57162
sim.sd.diff(5.182254,0.0962532,3.645762,0.07677712) # 2.446637

29.57162-(1.96*sqrt(2.446637)) # 26.50584
29.57162+(1.96*sqrt(2.446637)) # 32.6374




# BIKE PED CFR
svytotal(~died_any, subset(monInj, age < 20 & ped_cycl_mvc==1),
byvar=~yr, na.rm=T,multicore=T, se=T)
#                                SE
# _died_any_2006 599.9949 55.70074
# _died_any_2007 535.7119 56.13028
# _died_any_2008 602.6810 55.24830
# _died_any_2009 495.9098 49.63711
# _died_any_2010 467.9819 48.30128
# _died_any_2011 379.9875 44.06526
# _died_any_2012 448.5659 48.03025

# peds and cyclists visits
(totPedCyclCountYr<-svytotal(~count, subset(monInj, age < 20 & ped_cycl_mvc==1),
byvar=~yr, se=T, na.rm=T, multicore=T))
#                             SE
# _count_2006 71501.24 606.2437
# _count_2007 72661.04 599.9264
```

```
# _count_2008 71728.75 588.5382
# _count_2009 64714.18 572.4010
# _count_2010 64306.51 572.3481
# _count_2011 61370.59 544.0357
# _count_2012 60810.76 542.2433



# ped/bike CRF 2006
mean(rnorm(1000,599.9949, 55.70074)/
rnorm(1000,71501.24, 606.2437))*10000 # 83.66267
sd(rnorm(1000,599.9949, 55.70074)/
rnorm(1000,71501.24, 606.2437))*10000# 8.067546


# ped/bike CRF 2012
mean(rnorm(1000,448.5659, 48.03025)/
rnorm(1000,60810.76, 542.2433))*10000 # 73.6646
sd(rnorm(1000,448.5659, 48.03025)/
rnorm(1000,60810.76, 542.2433))*10000 # 7.737087


sim.mean.diff(83.66267,8.067546,73.6646,7.737087) # 11.83822 % decline
sim.sd.diff(83.66267,8.067546,73.6646,7.737087) # 14.0385

11.83822-(1.96*sqrt(14.0385)) # 4.494495
11.83822+(1.96*sqrt(14.0385)) # 19.18195
```

Level 1 or 2 trauma centers cared for 69.2% (se = 3.2) of all severely injured pediatric pedestrians and bicyclists in the US during the study period, with 15.0% (se=0.5) of all pediatric pedestrians and cyclists discharged from level 1 or 2 trauma centers were severely injured, compared with 4.0% (se=0.2) of all pedestrians and cyclists discharged from non-level 1 or 2 trauma centers.

The proportion of all severely injured pedestrians and cyclists discharged from level 1 or 2 trauma centers was 53.5% (se=2.0). The proportion of all pediatric pedestrian and cyclist deaths the occurred at level 1 or 2 trauma centers was 65.1% (se = 5.0).

6 Trauma Centers

```
svymean(~severe, se = T, na.rm = T, multicore = T, design = subset(monInj,
    age < 20 & ped_cycl_mvc == 1 & traumacenter == 1))
# SE severe 0.1497086 0.004991156

# proportion ped/cyclists seen in non-level 1 or 2 that are
```

```
# severely injured
svymean(~severe, se = T, na.rm = T, multicore = T, design = subset(monInj,
    age < 20 & ped_cycl_mvc == 1 & traumacenter == 0))
# SE severe 0.04049756 0.002024487


# severe injuries by trauma center (level 1 or 2) status peds
# and cyclists (used to calculate proportion of all severes
# seen in trauma centers)
svytotal(~severe, se = T, na.rm = T, multicore = T, design = subset(monInj,
    age < 20 & ped_cycl_mvc == 1 & traumacenter == 1))
# SE severe 20910 756.694

svytotal(~severe, se = T, na.rm = T, multicore = T, design = subset(monInj,
    age < 20 & ped_cycl_mvc == 1 & traumacenter == 0))
# SE severe 9330.122 476.4882



mean(rnorm(1000, 20910, 756.694)/(rnorm(1000, 20910, 756.694) +
    rnorm(1000, 9330.122, 476.4882)))  # 0.6925236
sd(rnorm(1000, 20910, 756.694)/(rnorm(1000, 20910, 756.694) +
    rnorm(1000, 9330.122, 476.4882)))  #  0.03230306



# severe injuries by trauma center status peds
svytotal(~severe, se = T, na.rm = T, multicore = T, design = subset(monInj,
    age < 20 & ped_mvc == 1 & traumacenter == 1))
# SE severe 15658.7 654.8179

svytotal(~severe, se = T, na.rm = T, multicore = T, design = subset(monInj,
    age < 20 & ped_mvc == 1 & traumacenter == 0))
# SE severe 6767.286 407.8183

# severe injuries by trauma center status cyclists
svytotal(~severe, se = T, na.rm = T, multicore = T, design = subset(monInj,
    age < 20 & cycl_mvc == 1 & traumacenter == 1))
# SE severe 5261.694 379.839

svytotal(~severe, se = T, na.rm = T, multicore = T, design = subset(monInj,
    age < 20 & cycl_mvc == 1 & traumacenter == 0))
# SE severe 2567.213 246.6548



# deaths by trauma center status peds and cyclists
```

```
svytotal(~died_any, se = T, na.rm = T, multicore = T, design = subset(monInj,
    age < 20 & ped_cycl_mvc == 1 & traumacenter == 1))
# SE died_any 1284.291 80.083

svytotal(~died_any, se = T, na.rm = T, multicore = T, design = subset(monInj,
    age < 20 & ped_cycl_mvc == 1 & traumacenter == 0))
# SE died_any 671.1545 61.69072


mean(rnorm(1000, 1284.291, 80.083)/(rnorm(1000, 1284.291, 80.083) +
    rnorm(1000, 671.1545, 61.69072)))  #0.6603256
sd(rnorm(1000, 1284.291, 80.083)/(rnorm(1000, 1284.291, 80.083) +
    rnorm(1000, 671.1545, 61.69072)))  #  0.05342848


# deaths by trauma center status peds
svytotal(~died_any, se = T, na.rm = T, multicore = T, design = subset(monInj,
    age < 20 & ped_mvc == 1 & traumacenter == 1))
# SE died_any 1066.253 72.93162

svytotal(~died_any, se = T, na.rm = T, multicore = T, design = subset(monInj,
    age < 20 & ped_mvc == 1 & traumacenter == 0))
# SE died_any 607.3471 58.16764


# deaths by trauma center status cyclists
svytotal(~died_any, se = T, na.rm = T, multicore = T, design = subset(monInj,
    age < 20 & cycl_mvc == 1 & traumacenter == 1))
# SE died_any 218.0381 33.07719

svytotal(~died_any, se = T, na.rm = T, multicore = T, design = subset(monInj,
    age < 20 & cycl_mvc == 1 & traumacenter == 0))
# SE died_any 63.80745 20.54843
```

7 Categories and Causes

The most common Barell matrix anatomic location designation for all pediatric pedestrians and cyclists discharged from US EDs during the study period was the lower extremity, which accounted for 45.0% (se = 0.8) of all discharges. The next most common locations were"other" head, face and neck injuries (17.8%, se = 0.4) and injuries to the upper extremities (12.5%, se = 0.3). Traumatic brain injuries (TBI) accounted for 6.7% (se = 0.2) of all discharges. By contrast, TBI was the most common injury location among pediatric pedestrians and cyclists who died, accounting for 55.5% (se = 14.1) injury location designations in that group. The unadjusted risk ratio for the association of TBI with fatality among injured

pediatric pedestrian and bicyclists was 8.4 (95% CI 5.5, 11.2).

```
# injury location ALL pedestrian and cyclists
(barell.all <- svytotal(~isrsite2descr, se = T, multicore = T,
    design = subset(monInj, age < 20 & ped_cycl_mvc == 1)))
# SE isrsite2descrLOWER_EXTREMITY 164258.69022 2269.94545
# isrsite2descrOTH_HEAD_FACE_NECK 64943.89553 1433.45654
# isrsite2descrOTHER___UNSPECIFIED 23263.94227 856.17594
# isrsite2descrSCI 28.37685 29.48528 isrsite2descrSYSTEM_WIDE
# 253.74778 93.22314 isrsite2descrTBI 24363.63891 900.92458
# isrsite2descrTORSO 32572.72470 1018.56526
# isrsite2descrUPPER_EXTREMITY 45645.27101 1195.67758
# isrsite2descrVCI_ 10129.73670 560.15422


tot <- rnorm(1000, 164258.69022, 2269.94545) + rnorm(1000, 64943.89553,
    1433.45654) + rnorm(1000, 23263.94227, 856.17594) + rnorm(1000,
    28.37685, 29.48528) + rnorm(1000, 253.74778, 93.22314) +
    rnorm(1000, 24363.63891, 900.92458) + rnorm(1000, 32572.7247,
    1018.56526) + rnorm(1000, 45645.27101, 1195.67758) + rnorm(1000,
    10129.7367, 560.15422)


low.ext <- rnorm(1000, 164258.69022, 2269.94545)
ent <- rnorm(1000, 64943.89553, 1433.45654)
upper.ext <- rnorm(1000, 45645.27101, 1195.67758)
tbi <- rnorm(1000, 24363.63891, 900.92458)

mean(low.ext/tot) * 100  #  44.95527
sd(low.ext/tot) * 100  # 0. 0.7539721

mean(ent/tot) * 100  # 17.77125
sd(ent/tot) * 100  # 0 0.4163906

mean(upper.ext/tot) * 100  # 12.47466
sd(upper.ext/tot) * 100  # 0.3443115


mean(tbi/tot) * 100  # 6.65274
sd(tbi/tot) * 100  # 0.2495455


# injury location all pedestrian and cyclists DIED
(barell.all <- svytotal(~isrsite2descr, se = T, multicore = T,
    design = subset(monInj, age < 20 & ped_cycl_mvc == 1 & died_any ==
        1)))
# SE isrsite2descrLOWER_EXTREMITY 40.573172 35.48164
# isrsite2descrOTH_HEAD_FACE_NECK 257.099589 96.58669
```

```
# isrsite2descrOTHER___UNSPECIFIED 209.409383 94.01797
# isrsite2descrSCI 0.000000 0.00000 isrsite2descrSYSTEM_WIDE
# 25.640256 32.40178 isrsite2descrTBI 897.864334 172.15149
# isrsite2descrTORSO 189.362946 77.08072
# isrsite2descrUPPER_EXTREMITY 15.458017 22.51615
# isrsite2descrVCI_ 4.820775 12.35639

tot.died <- rnorm(1000, 40.573172, 35.48164) + rnorm(1000, 257.099589,
    96.58669) + rnorm(1000, 209.409383, 94.01797) + rnorm(1000,
    0, 0) + rnorm(1000, 25.640256, 32.40178) + rnorm(1000, 897.864334,
    172.15149) + rnorm(1000, 189.362946, 77.08072) + rnorm(1000,
    15.458017, 22.51615) + rnorm(1000, 4.820775, 12.35639)

tbi.died <- rnorm(1000, 897.864334, 172.15149)

mean(tbi.died/tot.died) * 100  # 55.45005
sd(tbi.died/tot.died) * 100  #14.09559


# RISK RATIO TBI and DEATH
mean((tbi.died/tot.died)/(tbi/tot))  # 8.345666
sd((tbi.died/tot.died)/(tbi/tot))  # 2.145847

8.345666 - (1.96 * sqrt(2.145847))  # 5.474519
8.345666 + (1.96 * sqrt(2.145847))  # 11.21681





# injury location all pedestrian and cyclists didn't die
(barell.all <- svytotal(~isrsite2descr, se = T, multicore = T,
    design = subset(monInj, age < 20 & ped_cycl_mvc == 1 & died_any ==
        0)))
SE
# isrsite2descrLOWER_EXTREMITY 162194.06272 2255.63584
# isrsite2descrOTH_HEAD_FACE_NECK 64001.91155 1422.83819
# isrsite2descrOTHER___UNSPECIFIED 22801.89043 846.38085
# isrsite2descrSCI 28.37685 29.48732 isrsite2descrSYSTEM_WIDE
# 221.53215 86.04979 isrsite2descrTBI 23248.81526 880.58224
# isrsite2descrTORSO 31985.25578 1009.43060
# isrsite2descrUPPER_EXTREMITY 45093.48683 1188.04077
# isrsite2descrVCI_ 10001.48432 556.45860




# injury location all pedestrian
```

```r
(barell.all <- svytotal(~isrsite2descr, se = T, multicore = T,
    design = subset(monInj, age < 20 & ped_mvc == 1)))
SE
# isrsite2descrLOWER_EXTREMITY 125892.09930 1988.09518
# isrsite2descrOTH_HEAD_FACE_NECK 46620.36931 1216.90977
# isrsite2descrOTHER___UNSPECIFIED 16122.58461 717.39750
# isrsite2descrSCI 19.30733 24.14650 isrsite2descrSYSTEM_WIDE
# 198.95117 81.89841 isrsite2descrTBI 16832.37547 750.66674
# isrsite2descrTORSO 23948.77845 867.45704
# isrsite2descrUPPER_EXTREMITY 29744.18454 969.31498
# isrsite2descrVCI_ 6666.47200 456.24463


# percent tbi all pedestrians
tot.peds <- rnorm(1000, 125892.0993, 1988.09518) + rnorm(1000,
    46620.36931, 1216.90977) + rnorm(1000, 16122.58461, 717.3975) +
    rnorm(1000, 19.30733, 24.1465) + rnorm(1000, 198.95117, 81.89841) +
    rnorm(1000, 16832.37547, 750.66674) + rnorm(1000, 23948.77845,
    867.45704) + rnorm(1000, 29744.18454, 969.31498) + rnorm(1000,
    6666.472, 456.24463)

tbi.peds <- rnorm(1000, 16832.37547, 750.66674)


mean(tbi.peds/tot.peds) * 100  # 6.320036
sd(tbi.peds/tot.peds) * 100  # 0.2879068




# injury location PEDESTRIAN DIED
(barell.all <- svytotal(~isrsite2descr, se = T, multicore = T,
    design = subset(monInj, age < 20 & ped_mvc == 1 & died_any ==
        1)))
SE
# isrsite2descrLOWER_EXTREMITY 31.456050 30.67667
# isrsite2descrOTH_HEAD_FACE_NECK 233.404314 89.09163
# isrsite2descrOTHER___UNSPECIFIED 197.378305 92.36160
# isrsite2descrSCI 0.000000 0.00000 isrsite2descrSYSTEM_WIDE
# 16.181019 22.35600 isrsite2descrTBI 748.567745 156.02919
# isrsite2descrTORSO 169.005118 72.06635
# isrsite2descrUPPER_EXTREMITY 15.458017 22.51585
# isrsite2descrVCI_ 4.820775 12.35639




# injury location pedestrian didn't die
(barell.all <- svytotal(~isrsite2descr, se = T, multicore = T,
```

```
    design = subset(monInj, age < 20 & ped_mvc == 1 & died_any ==
        0)))
SE
# isrsite2descrLOWER_EXTREMITY 124223.89169 1975.02189
# isrsite2descrOTH_HEAD_FACE_NECK 45845.94022 1206.75543
# isrsite2descrOTHER___UNSPECIFIED 15739.76672 707.25566
# isrsite2descrSCI 19.30733 24.14826 isrsite2descrSYSTEM_WIDE
# 182.77015 78.78944 isrsite2descrTBI 15925.76446 731.00228
# isrsite2descrTORSO 23462.84493 858.65665
# isrsite2descrUPPER_EXTREMITY 29397.47423 963.25077
# isrsite2descrVCI_ 6588.34391 453.55361 injury location all
# cyclists
(barell.all <- svytotal(~isrsite2descr, se = T, multicore = T,
    design = subset(monInj, age < 20 & cycl_mvc == 1)))
SE
# isrsite2descrLOWER_EXTREMITY 38386.116492 1097.11837
# isrsite2descrOTH_HEAD_FACE_NECK 18333.877109 758.24498
# isrsite2descrOTHER___UNSPECIFIED 7153.539922 467.95510
# isrsite2descrSCI 9.069523 16.92124 isrsite2descrSYSTEM_WIDE
# 54.796615 44.53328 isrsite2descrTBI 7531.263441 498.32169
# isrsite2descrTORSO 8623.946253 533.97935
# isrsite2descrUPPER_EXTREMITY 15905.713100 700.35839
# isrsite2descrVCI_ 3463.264704 325.00104




# injury location CYCLISTS DIED
(barell.all <- svytotal(~isrsite2descr, se = T, multicore = T,
    design = subset(monInj, age < 20 & cycl_mvc == 1 & died_any ==
        1)))
SE
# isrsite2descrLOWER_EXTREMITY 9.117122 17.82833
# isrsite2descrOTH_HEAD_FACE_NECK 23.695275 37.30459
# isrsite2descrOTHER___UNSPECIFIED 12.031077 17.56754
# isrsite2descrSCI 0.000000 0.00000 isrsite2descrSYSTEM_WIDE
# 9.459237 23.45382 isrsite2descrTBI 149.296589 72.73731
# isrsite2descrTORSO 20.357829 27.34697
# isrsite2descrUPPER_EXTREMITY 0.000000 0.00000
# isrsite2descrVCI_ 0.000000 0.00000 injury location cyclists
# didn't die



# PROPORTION TBI CATEGORY using newly created indicator
# variable
```

```
# tbi all pedestrian and cyclists
svymean(~tbi, se = T, multicore = T, design = subset(monInj,
    age < 20 & ped_cycl_mvc == 1))
# SE tbi 0.06666567 0.002377668


# tbi all pedestrian and cyclists died
(svymean(~tbi, se = T, multicore = T, design = subset(monInj,
    age < 20 & ped_cycl_mvc == 1 & died_any == 1)))
# SE tbi 0.547402 0.07261212

# tbi all pedestrian and cyclists didn't die
(svymean(~tbi, se = T, multicore = T, design = subset(monInj,
    age < 20 & ped_cycl_mvc == 1 & died_any == 0)))
# SE tbi 0.06465605 0.002364548


# tbi all pedestrian
(svymean(~tbi, se = T, multicore = T, design = subset(monInj,
    age < 20 & ped_mvc == 1)))
# SE tbi 0.06326887 0.002726221

# tbi pedestrian died
(svymean(~tbi, se = T, multicore = T, design = subset(monInj,
    age < 20 & ped_mvc == 1 & died_any == 1)))
# SE tbi 0.5285483 0.07750631


# tbi pedestrian didn't die
(svymean(~tbi, se = T, multicore = T, design = subset(monInj,
    age < 20 & ped_mvc == 1 & died_any == 0)))
# SE tbi 0.06092812 0.002705462

# tbi all cyclists
(svymean(~tbi, se = T, multicore = T, design = subset(monInj,
    age < 20 & cycl_mvc == 1)))
# SE tbi 0.07572032 0.004808029


# tbi cyclists died
(svymean(~tbi, se = T, multicore = T, design = subset(monInj,
    age < 20 & cycl_mvc == 1 & died_any == 1)))
# SE tbi 0.6666302 0.2027402

# tbi cyclists didn't die
(svymean(~tbi, se = T, multicore = T, design = subset(monInj,
    age < 20 & cycl_mvc == 1 & died_any == 0)))
```

```
# SE tbi 0.07454443 0.004800812
```

In a series of regression regression models with robust covariances accounting for survey weighting and clustering, the unadjusted association between a pediatric pedestrian or cyclist injury with fatality, compared to all other pediatric emergency department discharges, was OR = 17.0 (95% CI 16.6, 17.3). The unadjusted association was higher for pedestrian injuries (OR=20.0, 95% CI 19.6, 20.5) than for cyclist injuries (OR= 8.3, 95% CI 7.9, 8.7). In a model comparing the association between pediatric pedestrian injury compared to cyclist injuries, controlling for potentially confounding factors and overall injury severity pediatric pedestrian injuries were twice as likely to be associated with a fatality compared to cyclist injuries (Table 1).

Variable	Odds Ratio (95% CI)
Pedestrian	2.4 (2.3, 2.6)
Age	0.7 (0.7, 0.7)
Female	0.9 (0.9, 1.0)
Severe	16.9 (15.9, 17.9)
TBI	6.3 (6.0, 6.6)
Trauma Center	0.9 (0.9, 1.0)

Table 1: Logistic Regression Association of Pedestrian vs. Cyclist Injury on the Risk of Emergency Department Fatality Controlling for Age, Gender, Injury Severity and Level 1 or 2 Trauma Center Status, with interaction term for Pedestrian Injury and TBI. US Emergency Department Discharges Pediatric Pedestrian and Bicyclist Injuries 2006-2012.

8 Regression

```
# bring in data set from monetDB
regDat <- as.data.frame(select(filter(NEDStab, age < 20), age,
    female, ped_cycl_mvc, ped_mvc, cycl_mvc, severe, tbi, traumacenter,
    died_any, discwt, neds_stratum))
# check number observations correct
str(regDat)  # 48,302,996

library(rms)
# prepare data distribution for robust covariances
d <- datadist(regDat)
options(datadist = "d")


# UNADJUSTED MODELS PED/BIKE VS. ALL OTHER VISITS

# unadjusted association of any ped or bike with fatality:
fatalOR1 <- lrm(died_any ~ ped_cycl_mvc, data = regDat, x = T,
```

```
    y = T, weight = discwt)
fatalOR1robust <- robcov(fatalOR1, cluster = regDat$neds_stratum)
summary(fatalOR1robust)
# Effects Response : died_any Factor Low High Diff. Effect
# S.E.  Lower 0.95 Upper 0.95 ped_cycl_mvc 0 1 1 2.8306
# 0.011533 2.808 2.8532 Odds Ratio 0 1 1 16.9560 NA 16.577
# 17.3440


# association of ped with fatality
fatalOR2 <- lrm(died_any ~ ped_mvc, data = regDat, x = T, y = T,
    weight = discwt)
fatalOR2robust <- robcov(fatalOR2, cluster = regDat$neds_stratum)
summary(fatalOR2robust)
# Effects Response : died_any Factor Low High Diff. Effect
# S.E.  Lower 0.95 Upper 0.95 ped_mvc 0 1 1 2.9976 0.012377
# 2.9734 3.0219 Odds Ratio 0 1 1 20.0380 NA 19.5580 20.5300

# association of bike with fatality
fatalOR3 <- lrm(died_any ~ cycl_mvc, data = regDat, x = T, y = T,
    weight = discwt)
fatalOR3robust <- robcov(fatalOR3, cluster = regDat$neds_stratum)
summary(fatalOR3robust)
# Effects Response : died_any Factor Low High Diff. Effect
# S.E.  Lower 0.95 Upper 0.95 cycl_mvc 0 1 1 2.1173 0.023673
# 2.0709 2.1637 Odds Ratio 0 1 1 8.3085 NA 7.9318 8.7031

# NB: both peds and or bike more fatal than any other ED
# visit type, but more than twice the risk unadjusted ped
# injury fatality compared to unadjusted bike fatality




# FULL MODEL PEDS VS. BIKES
d <- datadist(regDat2)
options(datadist = "d")

pedVbike1.rob <- robcov(lrm(died_any ~ ped_mvc + age + female +
    severe + tbi + traumacenter, data = regDat2, x = T, y = T,
    weight = discwt))
summary(pedVbike1.rob)
# Effects Response : died_any Factor Low High Diff. Effect
# S.E.  Lower 0.95 Upper 0.95 ped_mvc 0 1 1 0.889810 0.037932
# 0.815460 0.9641500 Odds Ratio 0 1 1 2.434700 NA 2.260200
# 2.6226000 age 9 16 7 -0.326350 0.010145 -0.346240
# -0.3064700 Odds Ratio 9 16 7 0.721550 NA 0.707340 0.7360400
```

```
# female 0 1 1 -0.034073 0.019395 -0.072087 0.0039406 Odds
# Ratio 0 1 1 0.966500 NA 0.930450 1.0039000 severe 0 1 1
# 2.827000 0.029094 2.770000 2.8840000 Odds Ratio 0 1 1
# 16.894000 NA 15.958000 17.8860000 tbi 0 1 1 1.837900
# 0.026877 1.785200 1.8906000 Odds Ratio 0 1 1 6.283400 NA
# 5.961000 6.6233000 traumacenter 0 1 1 -0.067938 0.028554
# -0.123900 -0.0119720 Odds Ratio 0 1 1 0.934320 NA 0.883470
# 0.9881000



# INTERACTION MODEL PEDS VS. BIKES

# robust model

pedVbike2.rob <- robcov(lrm(died_any ~ ped_mvc + age + female +
    severe + tbi + traumacenter + ped_mvc * tbi, data = regDat2,
    x = T, y = T, weight = discwt))


pedVbike2.rob
# Logistic Regression Model lrm(formula = died_any ~ ped_mvc
# + age + female + severe + tbi + traumacenter + ped_mvc *
# tbi, data = regDat2, x = T, y = T, weights = discwt) Sum of
# Weights by Response Category 0 1 307152.292 1286.801
# Frequencies of Missing Values Due to Each Variable died_any
# ped_mvc age female severe tbi traumacenter (weights) 1225 0
# 0 0 7373 22078 19995 0 Model Likelihood Discrimination Rank
# Discrim.  Ratio Test Indexes Indexes Obs 68319 LR chi2
# 4409.41 R2 0.270 C 0.867 0 68048 d.f.  7 g 1.136 Dxy 0.734
# 1 271 Pr(> chi2) <0.0001 gr 3.114 gamma 0.881 Sum of
# weights 308439.1 gp 0.006 tau-a 0.006 max |deriv| 2e-09
# Brier 0.004 Coef S.E.  Wald Z Pr(>|Z|) Intercept -7.3452
# 0.0636 -115.55 <0.0001 ped_mvc 1.2863 0.0626 20.55 <0.0001
# age -0.0464 0.0014 -32.22 <0.0001 female -0.0373 0.0193
# -1.93 0.0541 severe 2.8256 0.0291 97.21 <0.0001 tbi 2.4198
# 0.0744 32.55 <0.0001 traumacenter -0.0710 0.0285 -2.49
# 0.0127 ped_mvc * tbi -0.6646 0.0792 -8.39 <0.0001

exp(-0.6646)  # 0.5144793
exp(-0.6646 - 1.96 * sqrt(0.0792))  # 0.2963563
exp(-0.6646 + 1.96 * sqrt(0.0792))  # 0.8931443


summary(pedVbike2.rob)
# Effects Response : died_any Factor Low High Diff. Effect
# S.E.  Lower 0.95 Upper 0.95 ped_mvc 0 1 1 1.286300 0.062596
```

```
# 1.163600 1.40900000 Odds Ratio 0 1 1 3.619300 NA 3.201400
# 4.09170000 age 9 16 7 -0.325130 0.010089 -0.344900
# -0.30535000 Odds Ratio 9 16 7 0.722430 NA 0.708290
# 0.73686000 female 0 1 1 -0.037262 0.019345 -0.075178
# 0.00065379 Odds Ratio 0 1 1 0.963420 NA 0.927580 1.00070000
# severe 0 1 1 2.825600 0.029067 2.768700 2.88260000 Odds
# Ratio 0 1 1 16.872000 NA 15.937000 17.86100000 tbi 0 1 1
# 2.419800 0.074352 2.274100 2.56560000 Odds Ratio 0 1 1
# 11.244000 NA 9.719400 13.00800000 traumacenter 0 1 1
# -0.071013 0.028504 -0.126880 -0.01514600 Odds Ratio 0 1 1
# 0.931450 NA 0.880840 0.98497000 Adjusted to: ped_mvc=0
# tbi=0


summary(pedVbike2.rob, ped_mvc = 1, tbi = 1)
# Effects Response : died_any Factor Low High Diff. Effect
# S.E.  Lower 0.95 Upper 0.95 ped_mvc 0 1 1 0.621640 0.048916
# 0.525770 0.71751000 Odds Ratio 0 1 1 1.862000 NA 1.691800
# 2.04930000 age 9 16 7 -0.325130 0.010089 -0.344900
# -0.30535000 Odds Ratio 9 16 7 0.722430 NA 0.708290
# 0.73686000 female 0 1 1 -0.037262 0.019345 -0.075178
# 0.00065379 Odds Ratio 0 1 1 0.963420 NA 0.927580 1.00070000
# severe 0 1 1 2.825600 0.029067 2.768700 2.88260000 Odds
# Ratio 0 1 1 16.872000 NA 15.937000 17.86100000 tbi 0 1 1
# 1.755200 0.029045 1.698300 1.81210000 Odds Ratio 0 1 1
# 5.784600 NA 5.464500 6.12350000 traumacenter 0 1 1
# -0.071013 0.028504 -0.126880 -0.01514600 Odds Ratio 0 1 1
# 0.931450 NA 0.880840 0.98497000


summary(pedVbike2.rob, ped_mvc = 0, tbi = 1)
# Effects Response : died_any Factor Low High Diff. Effect
# S.E.  Lower 0.95 Upper 0.95 ped_mvc 0 1 1 0.621640 0.048916
# 0.525770 0.71751000 Odds Ratio 0 1 1 1.862000 NA 1.691800
# 2.04930000 age 9 16 7 -0.325130 0.010089 -0.344900
# -0.30535000 Odds Ratio 9 16 7 0.722430 NA 0.708290
# 0.73686000 female 0 1 1 -0.037262 0.019345 -0.075178
# 0.00065379 Odds Ratio 0 1 1 0.963420 NA 0.927580 1.00070000
# severe 0 1 1 2.825600 0.029067 2.768700 2.88260000 Odds
# Ratio 0 1 1 16.872000 NA 15.937000 17.86100000 tbi 0 1 1
# 2.419800 0.074352 2.274100 2.56560000 Odds Ratio 0 1 1
# 11.244000 NA 9.719400 13.00800000 traumacenter 0 1 1
# -0.071013 0.028504 -0.126880 -0.01514600 Odds Ratio 0 1 1
# 0.931450 NA 0.880840 0.98497000
```

8.1 Interaction on Additive Scale

When fatality rates per 100,000 population as outlined in table 2 were entered into an equation to test for inequality indicative of additive interaction for the presence of injury and alcohol or substance. The resulting calculation was positive for additive interaction: (4466.5 - 79.9)$(268.1-79.9)+(1968.3-79.9), indicatingthat 49.1\%(95\%CI 38.4, 59.4) oftheexcessmortalityinthegrou$

	TBI	No TBI
Pedestrian Injury	4466.5 (946.3)	268.1 (59.7)
Not Pedestrian Injury	1968.3 (1001.0)	79.9 (64.4)

Table 2: Contingency Table of Pre-Admission Fatality Rates per 100,000 population for Test of Additive Interaction Between Pedestrian Injury and TBI. US Emergency Department Discharges Pediatric Pedestrian and Bicyclist Injuries 2006-2012.

```
# CELL A numerator cell a: pedestrians with tbi who died
# _isrsite2descrTBI 748.567745 156.02919
num.a.sim <- rnorm(1000, 748.567745, 156.02919)
(num.a <- mean(num.a.sim))  # 750.1557
sd(num.a.sim)  # 156.1264

# denominator cell a: all pedestrians with tbi
# _isrsite2descrTBI 16832.37547 750.66674
denom.a.sim <- rnorm(1000, 16832.37547, 750.66674)
(denom.a <- mean(denom.a.sim))  # 16824.23
sd(denom.a.sim)  #  733.5134


# CELL B numerator cell b: pedestrians, not tbi, died
num.b.sim <- rnorm(1000, 31.45605, 30.67667) + rnorm(1000, 233.404314,
    89.09163) + rnorm(1000, 197.378305, 92.3616) + rnorm(1000,
    16.181019, 22.356) + rnorm(1000, 169.005118, 72.06635) +
    rnorm(1000, 15.458017, 22.51585) + rnorm(1000, 4.820775,
    12.35639)
(num.b <- mean(num.b.sim))  # 668.2694
sd(num.b.sim)  #  148.5901



# denominator cell b: all pedestrians not tbi
denom.b.sim <- rnorm(1000, 125892.0993, 1988.09518) + rnorm(1000,
    46620.36931, 1216.90977) + rnorm(1000, 16122.58461, 717.3975) +
    rnorm(1000, 19.30733, 24.1465) + rnorm(1000, 198.95117, 81.89841) +
    rnorm(1000, 23948.77845, 867.45704) + rnorm(1000, 29744.18454,
    969.31498) + rnorm(1000, 6666.472, 456.24463)
(denom.b <- mean(denom.b.sim))  # 249312.9
sd(denom.b.sim)  # 2816.72
```

```
# CELL C numerator cell c: cyclists with tbi who died
# isrsite2descrTBI 149.296589 72.73731
num.c.sim <- rnorm(1000, 149.296589, 72.73731)
(num.c <- mean(num.c.sim))  # 147.7841
sd(num.c.sim)  # 74.18056

# denominator cell c: all cyclists with tbi _isrsite2descrTBI
# 7531.263441 498.32169
denom.c.sim <- rnorm(1000, 7531.263441, 498.32169)
(denom.c <- mean(denom.c.sim))  # 7547.284
sd(denom.c.sim)  # 516.2133


# CELL D numerator cell d: cyclists without tbi who died
num.d.sim <- rnorm(1000, 9.117122, 17.82833) + rnorm(1000, 23.695275,
    37.30459) + rnorm(1000, 12.031077, 17.56754) + rnorm(1000,
    9.459237, 23.45382) + rnorm(1000, 20.357829, 27.34697)
(num.d <- mean(num.d.sim))  # 73.40336
sd(num.d.sim)  # 59.14791

# denominator cell d: all cyclists without tbi
denom.d.sim <- rnorm(1000, 38386.116492, 1097.11837) + rnorm(1000,
    18333.877109, 758.24498) + rnorm(1000, 7153.539922, 467.9551) +
    rnorm(1000, 9.069523, 16.92124) + rnorm(1000, 54.796615,
    44.53328) + rnorm(1000, 8623.946253, 533.97935) + rnorm(1000,
    15905.7131, 700.35839) + rnorm(1000, 3463.264704, 325.00104)
(denom.d <- mean(denom.d.sim))  # 91981.88
sd(denom.d.sim)  # 1686.602





# CALCULATION

# calculation for additive interaction (Risk_{peds * tbi} -
# Risk_unknown) = (Risk_peds - Risk_unknown) + (Risk_tbi -
# R_unknown)

# (rr_peds_tbi - rr_neither) = (rr_peds - rr_neither) +
# (rr_tbi - rr_neither)

rr_PED_TBI <- num.a.sim/denom.a.sim
rr_PED_notTBI <- num.b.sim/denom.b.sim
rr_notPED_TBI <- num.c.sim/denom.c.sim
```

```
rr_notPED_notTBI <- num.d.sim/denom.d.sim


mean(rr_PED_TBI) * 1e+05
mean(rr_PED_notTBI) * 1e+05
mean(rr_notPED_TBI) * 1e+05
mean(rr_notPED_notTBI) * 1e+05

# > mean(rr_PED_TBI)*100000 [1] 4466.462 >
# mean(rr_PED_notTBI)*100000 [1] 268.0793 >
# mean(rr_notPED_TBI)*100000 [1] 1968.272 >
# mean(rr_notPED_notTBI)*100000 [1] 79.8752


sd(rr_PED_TBI) * 1e+05
sd(rr_PED_notTBI) * 1e+05
sd(rr_notPED_TBI) * 1e+05
sd(rr_notPED_notTBI) * 1e+05

# > sd(rr_PED_TBI)*100000 [1] 946.3499 >
# sd(rr_PED_notTBI)*100000 [1] 59.67242 >
# sd(rr_notPED_TBI)*100000 [1] 1001.068 >
# sd(rr_notPED_notTBI)*100000 [1] 64.38894

L_side <- (rr_PED_TBI - rr_notPED_notTBI)
R_side <- (rr_PED_notTBI - rr_notPED_notTBI) + (rr_notPED_TBI -
    rr_notPED_notTBI)

mean(L_side)  # 0.04386587
mean(R_side)  #0.02076601

# L side greater, so additive interaction

# how much of excess mortality due to interaction?

prop.intx <- (L_side - R_side)/rr_PED_TBI

mean(prop.intx) * 100  # 49.13422
sd(prop.intx) * 100  # 27.53591
49.13422 - (1.96 * sqrt(27.53591))  # 38.84918
49.13422 + (1.96 * sqrt(27.53591))  # 59.41926
```

9 Appendix

9.1 Pedestrian and Cyclist Ecodes

The following ecodes were included in the definition of a pedestrian injured by being struck or involved in a collision with a moving motorized vehicle:

ECode	Description
E8002	railway accident involving collision with rolling stock and injuring pedestrian
E8012	railway accident involving collision with other object and injuring pedestrian
E8022	railway accident involving derailment without antecedent collision injuring pedestrian
E8032	railway accident involving explosion, fire, or burning injuring pedestrian
E8042	fall in, on, or from railway train injuring pedestrian
E8052	pedestrian hit by rolling stock
E8062	other specified railway accident injuring pedestrian
E8072	railway accident of unspecified nature injuring pedestrian
E8107	motor vehicle traffic accident involving collision with train injuring pedestrian
E8117	motor vehicle traffic accident involving re-entrant collision with another motor vehicle injuring pedestrian
E8127	other motor vehicle traffic accident involving collision with motor vehicle injuring pedestrian
E8137	motor vehicle traffic accident involving collision with other vehicle injuring pedestrian
E8147	motor vehicle traffic accident involving collision with pedestrian injuring pedestrian
E8157	other motor vehicle traffic accident involving collision on the highway injuring pedestrian
E8167	motor vehicle traffic accident due to loss of control, without collision on the highway, injuring pedestrian
E8177	noncollision motor vehicle traffic accident while boarding or alighting injuring pedestrian
E8187	other noncollision motor vehicle traffic accident injuring pedestrian
E8197	motor vehicle traffic accident of unspecified nature injuring pedestrian
E8207	nontraffic accident involving motor-driven snow vehicle injuring pedestrian
E8217	nontraffic accident involving other off-road motor vehicle injuring pedestrian
E8227	other motor vehicle nontraffic accident involving collision with moving object injuring pedestrian
E8237	other motor vehicle nontraffic accident involving collision with stationary object injuring pedestrian
E8247	other motor vehicle nontraffic accident while boarding and alighting injuring pedestrian
E8257	other motor vehicle nontraffic accident of other and unspecified nature injuring pedestrian
E8260	pedal cycle accident injuring pedestrian
E8270	animal-drawn vehicle accident injuring pedestrian
E8280	accident involving animal being ridden injuring pedestrian
E8290	other road vehicle accidents injuring pedestrian

Table 3: Pedestrian-Injury Related Ecodes Included in the Definition of a Pedestrian Injured by Being Struck or Involved in a Collision With a Moving Motorized Vehicle

The following ecodes were included in the definition of a cyclist injured by being struck or involved in a collision with a moving motorized vehicle:

The following ecodes were excluded from the definition of pedestrians because they specified injuries to drivers or others due to collisions with pedestrians.

E8140 MV COLL W PEDEST-DRIVER (Motor Vehicle Traffic Accident Involving Collision With Pedestrian Injuring Driver Of Motor Vehicle Other Than Motorcycle)

E8141 MV COLL W PEDEST-PASNGR (Motor Vehicle Traffic Accident Involving Collision With Pedestrian Injuring Passenger In Motor Vehicle Other Than Motorcycle)

ECode	Description
E8003	railway accident involving collision with rolling stock and injuring pedal cyclist
E8023	railway accident involving derailment without antecedent collision injuring pedal cyclist
E8033	railway accident involving explosion, fire, or burning injuring pedal cyclist
E8043	fall in, on, or from railway train injuring pedal cyclist
E8053	pedal cyclist hit by rolling stock
E8063	other specified railway accident injuring pedal cyclist
E8073	railway accident of unspecified nature injuring pedal cyclist
E8106	motor vehicle traffic accident involving collision with train injuring pedal cyclist
E8116	motor vehicle traffic accident involving re entrant collision with another motor vehicle injuring pedal cyclist
E8126	other motor vehicle traffic accident involving collision with motor vehicle injuring pedal cyclist
E8136	motor vehicle traffic accident involving collision with other vehicle injuring pedal cyclist
E8146	motor vehicle traffic accident involving collision with pedestrian injuring pedal cyclist
E8156	other motor vehicle traffic accident involving collision on the highway injuring pedal cyclist
E8166	motor vehicle traffic accident due to loss of control, without collision on the highway, injuring pedal cyclist
E8176	noncollision motor vehicle traffic accident while boarding or alighting injuring pedal cyclist
E8186	other noncollision motor vehicle traffic accident injuring pedal cyclist
E8196	motor vehicle traffic accident of unspecified nature injuring pedal cyclist
E8206	nontraffic accident involving motor driven snow vehicle injuring pedal cyclist
E8216	nontraffic accident involving other off road motor vehicle injuring pedal cyclist
E8226	other motor vehicle nontraffic accident involving collision with moving object injuring pedal cyclist
E8236	other motor vehicle nontraffic accident involving collision with stationary object injuring pedal cyclist
E8246	other motor vehicle nontraffic accident while boarding and alighting injuring pedal cyclist
E8256	other motor vehicle nontraffic accident of other and unspecified nature injuring pedal cyclist

Table 4: Bicycle-Injury Related Ecodes Included in the Definition of a Cyclist Injured by Being Struck or Involved in a Collision With a Moving Motorized Vehicle

E8142 MV COLL W PEDEST-MOTCYCL (Motor vehicle traffic accident involving collision with pedestrian injuring motorcyclist)

E8144 MV COLL W PEDEST-ST CAR (Motor Vehicle Traffic Accident Involving Collision With Pedestrian Injuring Occupant Of Streetcar)

The following ecodes were excluded from the definition of injured cyclists because they either did not explicitly involve a motorized moving vehicle or specified injuries to someone other than the cyclist.

E8261 PED CYCL ACC-PED CYCLIST (Pedal Cycle Accident Injuring Pedal Cyclist)

E8262 PED CYCLE ACC-ANIM RIDER (Pedal cycle accident injuring rider of animal)

E8263 PED CYC ACC-OCC ANIM VEH (Pedal Cycle Accident Injuring Occupant Of Animal-Drawn Vehicle)

E8264 PED CYCLE ACC-OCC ST CAR (Pedal cycle accident injuring pedal cyclist)

E8268 PED CYCLE ACC-PERS NEC (Pedal Cycle Accident Injuring Other Specified Person)

E8269 PED CYCLE ACC-PERS NOS (Pedal Cycle Accident Injuring Unspecified Person)

Note that the large majority of all bike-related ecodes (549,248 of the total 632,551 bike-related ecodes) did not specify the involvement of a motorized moving vehicles, and were

excluded.

```
# pedestrian ecodes for injuries to drivers
dbGetQuery(neds, "select sum(count) from neds_0612 where
ecode1 in  ('E8140', 'E8141', 'E8142', 'E8144') OR
ecode2 in  ('E8140', 'E8141', 'E8142', 'E8144') OR
ecode3 in  ('E8140', 'E8141', 'E8142', 'E8144') OR
ecode4 in  ('E8140', 'E8141', 'E8142', 'E8144')
;")  # 4521

# pediatric pedestrian ecodes for injuries to drivers
dbGetQuery(neds, "select sum(count) from neds_0612 where
(ecode1 in  ('E8140', 'E8141', 'E8142', 'E8144') OR
ecode2 in  ('E8140', 'E8141', 'E8142', 'E8144') OR
ecode3 in  ('E8140', 'E8141', 'E8142', 'E8144') OR
ecode4 in  ('E8140', 'E8141', 'E8142', 'E8144'))
AND age <20
;")  # 943
```

9.2 R Syntax to Create Pedestrian and Cyclist Variables

```
# ecode is type clob, variable length character print first
# 10 ecodes
dbGetQuery(neds, "SELECT ecode1 FROM neds_0612 LIMIT 10;")

# ecode1 1 E927 2 E8259 3 E8859 4 E9203 5 E9208 6 E918 7
# E9241 8 E9174 9 E8859 10 E9179


# I. Pedstrian Motor Vehicle Injury Variable

# count up all pedestrian codes 260,365 entries
dbGetQuery(neds, [1174 chars quoted with '"'])

# create pedestrian variable
dbGetQuery(neds, "alter table neds_0612 add ped_mvc int default 0;")
dbSendQuery(neds, [1169 chars quoted with '"'])

dbGetQuery(neds, "select sum(count) from neds_0612 where ped_mvc=1;")  # 260,365




# II. Cyclist Motor Vehicle Injury Variable

# count up all cyclist codes 83,303 entries
dbGetQuery(neds, "select sum(count) from neds_0612 where
```

```
 ecode1 in   ('E8003', 'E8023', 'E8033', 'E8043', 'E8053', 'E8063', 'E8073', 'E8106',
 'E8116', 'E8126', 'E8136', 'E8146', 'E8156', 'E8166', 'E8176', 'E8186', 'E8196', 'E8206',
 'E8216', 'E8226', 'E8236', 'E8246', 'E8256') OR
 ecode2 in   ('E8003', 'E8023', 'E8033', 'E8043', 'E8053', 'E8063', 'E8073', 'E8106',
 'E8116', 'E8126', 'E8136', 'E8146', 'E8156', 'E8166', 'E8176', 'E8186', 'E8196', 'E8206',
 'E8216', 'E8226', 'E8236', 'E8246', 'E8256') OR
 ecode3 in   ('E8003', 'E8023', 'E8033', 'E8043', 'E8053', 'E8063', 'E8073', 'E8106',
 'E8116', 'E8126', 'E8136', 'E8146', 'E8156', 'E8166', 'E8176', 'E8186', 'E8196', 'E8206',
 'E8216', 'E8226', 'E8236', 'E8246', 'E8256') OR
 ecode4 in   ('E8003', 'E8023', 'E8033', 'E8043', 'E8053', 'E8063', 'E8073', 'E8106',
 'E8116', 'E8126', 'E8136', 'E8146', 'E8156', 'E8166', 'E8176', 'E8186', 'E8196', 'E8206',
 'E8216', 'E8226', 'E8236', 'E8246', 'E8256')
 ;")

# create cyclist variable
dbGetQuery(neds, "alter table neds_0612 add cycl_mvc int default 0;")
dbSendQuery(neds, "update neds_0612 set cycl_mvc=1 where
 ecode1 in   ('E8003', 'E8023', 'E8033', 'E8043', 'E8053', 'E8063', 'E8073', 'E8106',
 'E8116', 'E8126', 'E8136', 'E8146', 'E8156', 'E8166', 'E8176', 'E8186', 'E8196',
 'E8206', 'E8216', 'E8226', 'E8236', 'E8246', 'E8256') OR
 ecode2 in   ('E8003', 'E8023', 'E8033', 'E8043', 'E8053', 'E8063', 'E8073', 'E8106',
 'E8116', 'E8126', 'E8136', 'E8146', 'E8156', 'E8166', 'E8176', 'E8186', 'E8196',
 'E8206', 'E8216', 'E8226', 'E8236', 'E8246', 'E8256') OR
 ecode3 in   ('E8003', 'E8023', 'E8033', 'E8043', 'E8053', 'E8063', 'E8073', 'E8106',
 'E8116', 'E8126', 'E8136', 'E8146', 'E8156', 'E8166', 'E8176', 'E8186', 'E8196',
 'E8206', 'E8216', 'E8226', 'E8236', 'E8246', 'E8256') OR
 ecode4 in   ('E8003', 'E8023', 'E8033', 'E8043', 'E8053', 'E8063', 'E8073',
 'E8106', 'E8116', 'E8126', 'E8136', 'E8146', 'E8156', 'E8166', 'E8176', 'E8186',
 'E8196', 'E8206', 'E8216', 'E8226', 'E8236', 'E8246', 'E8256')
 ;")

dbGetQuery(neds, "select sum(count) from neds_0612 where cycl_mvc=1;")  # 83,303



# III. Pedestrian or Cyclist Variable count up peds and
# cyclists
dbGetQuery(neds, "select sum(count) from neds_0612 where ped_mvc=1 or cycl_mvc=1;")  # 343,615 (NB: 83[illegible]
# create ped_cyclist
dbGetQuery(neds, "alter table neds_0612 add ped_cycl_mvc int default 0;")
dbSendQuery(neds, "update neds_0612 set ped_cycl_mvc=1 where ped_mvc=1 or cycl_mvc=1;")

dbGetQuery(neds, "select sum(count) from neds_0612 where ped_cycl_mvc=1;")  # 343,615
```
